# Alveolar macrophages initiate the spatially targeted recruitment of neutrophils after nanoparticle inhalation

**DOI:** 10.1126/sciadv.adx8586

**Published:** 2025-11-07

**Authors:** Qiongliang Liu, Lin Yang, Chenxi Li, Lianyong Han, Juliet Wilmot, Guo Yang, Riccardo Leinardi, Qiaoxia Zhou, Andreas Schröppel, David Kutschke, Judith Secklehner, Ali Önder Yldirim, François Huaux, Dagmar Zeuschner, Leo M. Carlin, Markus Sperandio, Otmar Schmid, Tobias Stoeger, Markus Rehberg

**Affiliations:** ^1^ Institute of Lung Health and Immunity (LHI), Comprehensive Pneumology Center (CPC), Helmholtz Center Munich, Member of the German Center for Lung Research (DZL), Munich, Germany.; ^2^ Department of Thoracic Surgery, Shanghai General Hospital, Shanghai Jiao Tong University School of Medicine, Shanghai 200080, China.; ^3^ Louvain Center for Toxicology and Applied Pharmacology (LTAP), Institut de Recherche Expérimental et Clinique (IREC), Université Catholique de Louvain, Louvain-la-Neuve, Belgium.; ^4^ Cancer Research UK Scotland Institute, Glasgow, UK.; ^5^ School of Cancer Sciences, University of Glasgow, Glasgow, UK.; ^6^ Electron Microscopy Facility, Max Planck Institute for Molecular Biomedicine, Muenster, Germany.; ^7^ Walter Brendel Centre of Experimental Medicine, Biomedical Center, Institute of Cardiovascular Physiology and Pathophysiology, Ludwig-Maximilians-Universität München, Planegg-Martinsried, Germany.

## Abstract

Lung resident immune cells are essential for initiating defenses against inhaled air pollutants, including nanoparticles (NPs), which contribute to pulmonary disease progression. Here, lung intravital microscopy was used to examine the pulmonary innate immune responses in mice, during acute aerosol exposure to carbon NPs, a common environmental pollutant, or fluorescent quantum dot NPs. We found that inhaled NPs triggered rapid, neutrophil recruitment, localized to alveolar NP deposition hotspots, orchestrated by alveolar macrophages (AMs) through both their motility and phagocytic activity. AM motility inhibition in the alveoli via intercellular adhesion molecule–1/LFA-1 blockade reduced neutrophil recruitment, as did impaired AM phagocytosis through C5a receptor 1/Fc-γ receptor I inhibition or by stealth NP surfaces. In addition, cellular degranulation inhibition indicated the importance of spatially focused cytokine release in neutrophil recruitment. Collectively, our study elucidates AM-epithelial interactions as a critical key event for NPs triggered neutrophilia, with AM motility and phagocytosis driving recruitment and site-specific immune responses in the alveolar microenvironment.

## Teaser

Intravital imaging shows how inhaled nanoparticles activate alveolar macrophages to trigger the lung innate immune response.

## INTRODUCTION

Respiratory and cardiovascular diseases are closely associated with air pollution, particularly due to airborne particulate matter (PM), including fine particles (PM 2.5 with a diameter of ≤2.5 μm) and ultrafine particles or nanoparticles (NPs) (PM 0.1 with a diameter of ≤0.1 μm), which contribute to increased mortality rates (*1*). Because of their nanoscale size, these particles are highly efficient at penetrating deep into the lungs, depositing in the fragile alveolar regions, and posing a greater health risk compared to larger particles due to their high surface area–to–mass ratio (*2*). Inhalation of manufactured and incidental nanomaterials and NPs (1 to 100 nm) also largely contributes to acute respiratory distress syndrome and the development of chronic lung diseases (*3*, *4*). Acute and transient pneumonitis is the most common reaction that can be triggered in vivo by almost any inhaled nanomaterial, depending on the dose delivered to the respiratory barrier. Although the most biologically relevant dose metric for the acute inflammatory response to inhaled, poorly soluble, low-toxicity particles has been identified as the surface area of deposited NPs (*5*), there is still a lack of understanding of the underlying molecular processes triggered by inhaled substances such as NPs, which is crucial for the development of effective preventive and therapeutic strategies.

Carbonaceous NPs represent an important proportion of ambient, urban particles, and pulmonary exposure to low doses has been shown to induce rapid proinflammatory responses in the lung including the release of proinflammatory cytokines (*6*–*8*). It is well known that tissue-resident macrophages and attracted polymorphonuclear neutrophils, both important parts of the innate immune system, act rapidly and nonspecifically to protect the body upon any harmful lung exposures (e.g., pathogens or carbon-based particles) (*9*–*12*). Although the immune response elicited to pathogens via specific receptors is well understood, a comprehensive understanding of the early events that trigger the innate immune response following NP inhalation remains lacking, despite extensive research on the respiratory toxicity of NPs.

Innate immune cell dynamics, including surveillance, motility, and phagocytosis, are crucial for the rapid detection and elimination of harmful NPs, ensuring an immediate and effective immune response (*13*). These dynamic behaviors also coordinate the recruitment and activation of other immune cells, shaping the spatial and temporal regulation of inflammation to protect and maintain alveolar homeostasis (*14*, *15*). Our current understanding of the mode of action for poorly soluble particles of low toxicity describes particle surface and particularly its pro-oxidative state to drive and determine their inflammatory potential (*16*, *17*). However, experimental approaches usually lack the ability to capture particle deposition and in vivo biodistribution of NPs, as well as the dynamic cellular cycles that precede and trigger specific biological effects, thereby initiating the inflammatory response in the early phases of NP exposure. This limitation might contribute to the fact that the sequence of events, particularly the interplay of tissue-resident macrophages and alveolar epithelial cells (AECs) in triggering spatially and temporally defined neutrophil recruitment, is still unclear.

Alveolar macrophages (AMs), situated in close contact with the lung epithelium in the alveolar space, play a crucial role as the first line of defense against inhaled pathogens (*18*) and air pollution–derived particles. AMs have been linked to the initiation of lung inflammation caused by air pollution particles (*19*); however, sensing of microbial components such as endotoxin by specific pattern recognition receptors, such as Toll-like receptors (TLRs), might modulate the inflammatory responses to ambient PM, and the role of AMs as initiating cells for sterile and pyrogen-free particles might be different. Previous animal studies have shown that AMs show no transcriptional activation upon carbon NP (CNP) exposure and TLR2 and TLR4 are not involved in the inflammatory response observed within 24 hours (*7*, *20*). To resolve these contradictions, it is essential to investigate the alveolus during particle inhalation and study the ensuing cellular events in real time.

The dense capillary network of the lung maximizes the direct contact between air and blood, enhancing oxygen exchange efficiency (*21*). The enormous surface of the air-blood barrier, however, also increases susceptibility to inhaled infectious pathogens or NPs. Neutrophils not only are the first responders recruited to sites of injury or infection, marking the onset of acute inflammation, but also play a role in its resolution. Once recruited to the lung, neutrophils amplify the immune response by infiltrating the alveoli, releasing chemokines, and activating downstream signaling pathways (*21*–*23*).

Lung intravital microscopy (L-IVM) has been successfully used to study neutrophil dynamics in microvessels in the alveolar region of murine lungs (*24*, *25*) and has recently been extended to the analysis of AM functions, whereby AM crawling in and between alveoli has been demonstrated (*26*). However, the spatiotemporal dynamics of AM activity and NP-induced neutrophil recruitment from the pulmonary microvasculature to the alveolar compartment during the early phase of NP-induced lung response remains elusive. In addition, the molecular pathways underlying AM adhesion, migration, and NP recognition triggered by inhaled NPs are yet unknown.

Therefore, we used L-IVM combined with a ventilator-assisted aerosol delivery system. Fluorescent quantum dot (QD) NPs were used to monitor particle deposition and uptake, and carbon black (soot) NPs (CNPs) were used as a surrogate for ambient particles. This approach allowed concomitant real-time observation of NP-aerosol deposition on the alveolar surface of the lung, as well as the corresponding behavior of lung-resident phagocytes and the recruitment of leukocytes from the pulmonary microcirculation. Moreover, we investigated the molecular mechanisms governing the innate immune response by using various functional blocking antibodies/inhibitors and by altering the surface properties of inhaled NPs. The presented data indicate a close relation between AM activity (phagocytosis and migration) and the rapid and NP site-specific recruitment of neutrophils during the early phase of NP inhalation (<60 min), demonstrating a specific role of AMs in mounting the immune response upon NP inhalation.

## RESULTS

**Fig. 1.**
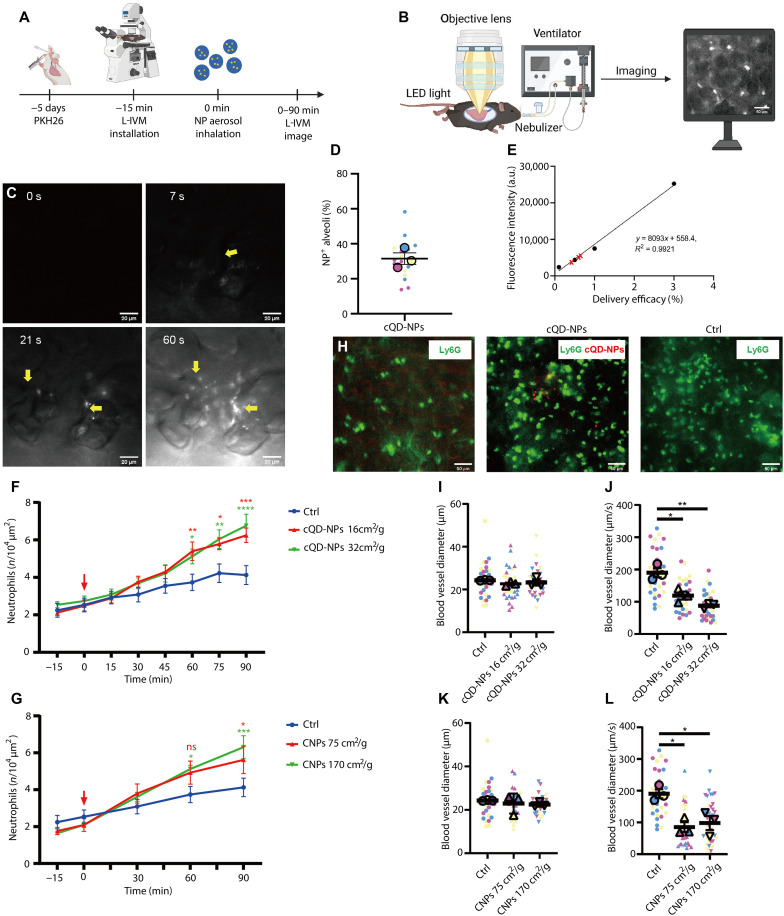
NP inhalation causes rapid recruitment of neutrophils in the pulmonary microcirculation, with associated hemodynamic changes. (**A**) Schematic of NP inhalation experiments. (**B**) L-IVM in ventilated mice using a nebulizer for NP aerosol inhalation. (**C**) Inhaled cQD-NPs appeared rapidly as fluorescent spots in single alveoli (yellow arrows), indicating aerosol droplets. Spot count and fluorescence intensity increased over time. Scale bars, 50 μm. (**D**) Five minutes after cQD-NP inhalation, L-IVM quantified cQD-NP-positive alveoli (16 cm^2^/g, NP surface area per mass lung) across seven fields of view (FOVs) per mouse (20 to 30 alveoli/FOV, *n* = 3). Mean values per mouse are shown as larger same-colored circles. (**E**) Lung delivery efficacy of cQD-NPs was calculated from a fluorescence standard curve (*R*^2^ > 0.99) using spiked lung homogenates and normalized to the aerosolized dose (black circles). Red marks show cQD-NP fluorescence from three independent inhalations. a.u., arbitrary units. (**F**) Quantitative analysis of neutrophils after inhaling two cQD-NP doses. NPs inhaled at 0 min (red arrow). Inhalation typically lasted 1 min for QDs (*n* = 6 mice per group). (**G**) Quantitative analysis of neutrophils after inhaling two CNP doses. NPs inhaled at 0 min (red arrow), Inhalation typically lasted 5 min for CNPs (*n* = 4 to 6 mice per group). (**H**) L-IVM images of neutrophils [anti-Ly6G monoclonal antibodies (mAbs), intravenously; green] at 90 min postinhalation of vehicle (control), cQD-NPs (16 cm^2^/g; red), and CNPs (75 cm^2^/g). Scale bars, 50 μm. (**I** and **K**) Alveolar microvessel diameters are unaffected by cQD-NP and CNP inhalation. Each data point shows one microvessel; larger circles/triangles indicate the mean per mouse (*n* = 3). (**J** and **L**) Blood flow velocities in pulmonary microcirculation decrease after cQD-NP and CNP exposure, respectively. Velocities were tracked via fluorescent beads (1 μm) at 90 min; 10 beads per mouse (*n* = 3). Data are shown as means ± SEM; [(F), (G), and (I) to (L)]: One-way analysis of variance (ANOVA). n.s., *P* ≥ 0.05; *P < 0.05, **P < 0.01, ***P < 0.001, and ****P < 0.0001.

To observe, in real time, the deposition of inhaled particles in the lungs of living mice and simultaneously study the immune response in the alveolar region of the lungs, we combined a ventilator-assisted aerosol inhalation system with L-IVM (Fig. 1, A and B) (*27*). We used fluorescent carboxyl-QD (cQD)–NPs (diameter, 15 nm), to characterize NP lung deposition dynamics, which have previously been extensively used to study adverse in vivo effects and cellular uptake of NPs (*28*–*31*). The nebulizer used for ventilator-assisted aerosol delivery generates aerosol droplets (2.5 to 4.0 μm) containing NP suspensions. The NP doses (cQD-NPs and in the following CNPs) were chosen, such that they induce a comparable level of neutrophil recruitment in the lung.

NPs reached peripheral alveoli seconds after the onset of inhalation and were immediately detected as distinct fluorescent spots by L-IVM (Fig. 1C). Notably, not all alveoli in the field of views exhibited deposited cQD-NPs. At the administered dose of 16 cm^2^/g (geometric NP surface area/mass lung) used in this study, 31.47 ± 2.2% of alveoli in the recorded field of views received cQD-NPs (Fig. 1D). To quantify the lung deposited cQD-NP-NP dose after ventilator-assisted nebulization of 20 μl of a 4 μM cQD-NP suspension, mice were euthanized immediately after NP application, and the QD fluorescence in the lung tissue was quantified via spectrofluorometry in lung homogenate. The cQD-NP deposition efficacy was determined as 0.53 ± 0.10% (means ± SEM; Fig. 1E).

To investigate the potential inflammatory effects of cQD-NP-NPs in the alveolar region, we used L-IVM to observe and quantify neutrophil recruitment upon ventilator-assisted cQD-NP inhalation in pulmonary microvessels. For this, neutrophils were immunolabeled in the vascular compartment by intravenously injected fluorescent anti-Ly6G antibodies 10 min before imaging (Fig. 1B). Already at 30 min upon inhalation of a lung-deposited dose of cQD-NPs (16 cm^2^/g), an increased number of neutrophils, as compared to the control group, was determined in the observation areas, which became significant at 60 min. Increasing the deposited dose (from 16 to 32 cm^2^/g) of cQD-NPs did not cause a significant difference in the numbers of recruited neutrophils, as determined by semiautomated quantitative detection of fluorescent neutrophils (Fig. 1F). To determine whether leukocyte recruitment dynamics obtained for cQD-NPs are comparable to the inflammatory response induced by inhalation of a bioequivalent dose of CNPs, a common component of urban air pollution (*7*), we exposed mice to calculated deposited CNP doses of 75 and 170 cm^2^/g by ventilator-assisted inhalation. Sixty minutes after CNP inhalation, a large number of neutrophils accumulated in the lungs of mice receiving CNP (75 or 170 cm^2^/g) via inhalation (Fig. 1, G and H), with cell counts comparable to those observed in cQD-NP-exposed animals (Fig. 1, F and H). From Fig. 1 (F and G), it is apparent that the onset of neutrophil recruitment is independent of dose, while the rate of recruitment may be dose dependent at later time points, which is seen as a nonsignificant trend in the respective 90-min data. The detected numbers of neutrophils at 90 min after cQD-NP and CNP inhalation correspond to neutrophil amounts previously detected by our group, 60 min after lung instillation of endotoxin [0.1 μg of lipopolysaccharide (LPS)] (*32*).

Inflammation can potentially cause changes in pulmonary microvascular hemodynamics that affect blood flow velocity and shear rates (*25*). To assess the impact of NP inhalation on the alveolar microcirculation, we measured the blood flow velocities in the lungs of cQD-NP-exposed mice by tracing of intravenously injected fluorescent microbeads. Blood flow velocities were decreased at 90 min after inhalation of both cQD-NPs and CNPs (Fig. 1, J and L). Pulmonary microvessel diameters were not altered among the experimental groups (Fig. 1, I and K), excluding local vasoconstriction or vasodilation as a cause for NP inhalation–induced blood flow velocity alterations. Together, inhalation of cQD-NPs and CNPs caused rapid neutrophil recruitment and blood flow velocity reduction in the pulmonary microcirculation.

Differential cell analysis in bronchoalveolar lavage (BAL) fluid was applied to further define inflammatory responses caused by cQD-NP or CNP inhalation (cQD-NP, 16 cm^2^/g; CNP, 170 cm^2^/g). A low number of neutrophils was detected 2 hours after inhalation of cQD-NPs, whereas neutrophils were almost undetectable in control animals (fig. S1A). Likewise, in the CNP-exposed group, neutrophil numbers were elevated, although not significant (fig. S1B). Compared with the control group, the amounts of total cells, AMs, and lymphocytes were similar in the cQD-NP- and CNP-exposed groups (fig. S1, A and B). At 24 hours upon cQD-NP and CNP inhalation, a substantial influx of neutrophils (5 to 10% of total BAL cells) into the airspace occurred, contrasting with almost undetectable levels in BAL of control mice (fig. S1, C and D). Together, BAL analysis confirmed an acute inflammatory response induced by cQD-NPs and CNPs, marked by a notable accumulation of neutrophils at 2 and 24 hours after exposure.

### Neutrophils are recruited in pulmonary microvessels in close proximity to the sites of cQD-NP-NP deposition

**Fig. 2.**
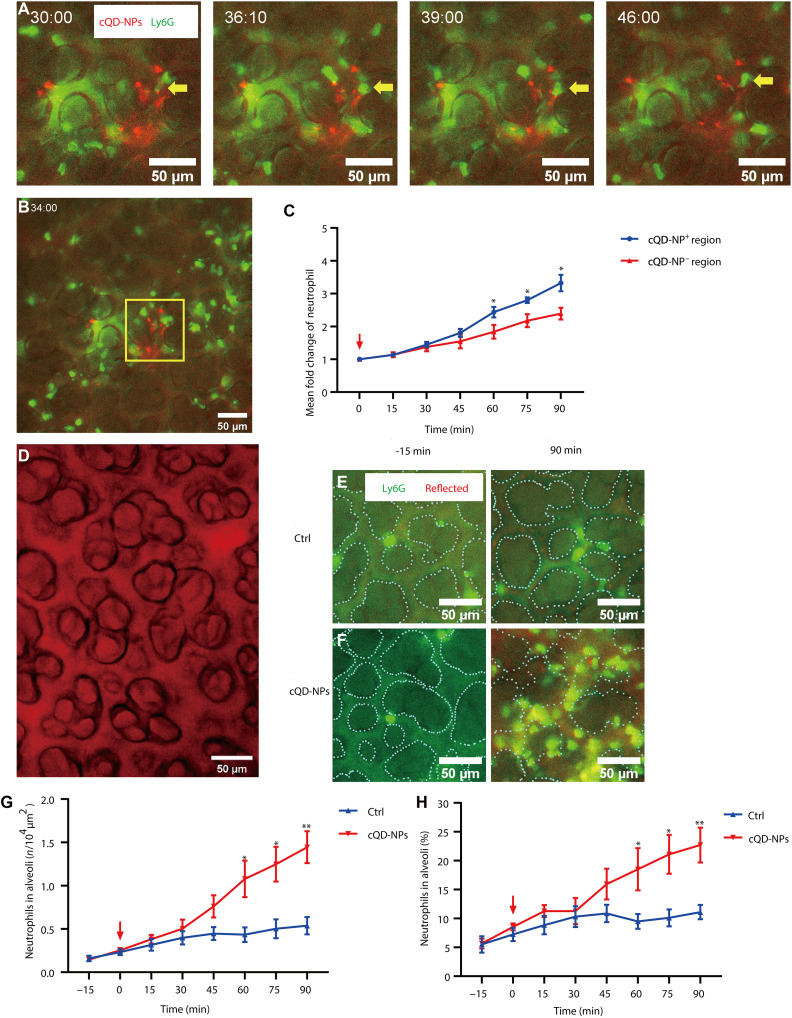
Neutrophil recruitment and infiltration into the alveoli in response to inhaled NPs occur in close proximity to the site of NP deposition. (**A**) Time lapse of a cQD-NP-probing neutrophil (yellow arrow) in a cQD-NP-abundant area. cQD-NP-exposed (exposure dose, 16 cm^2^/g; red) mice intravenously injected with anti-Ly6G to label neutrophils (green), Scale bars, 50 μm. (**B**) Large numbers of neutrophils accumulate in alveolar regions close to deposited cQD-NPs (0.01-mm^2^ yellow square; 100 μm in side length). Scale bar, 50 μm. (**C**) Neutrophil counts during L-IVM were compared between cQD-NP-rich and cQD-NP-poor regions. Counts in cQD-NP-rich areas were analyzed in 0.01-mm^2^ squares centered on hotspots shown in (B). Data are mean fold change from baseline ± SEM (*n* = 3 mice per group, seven FOVs per mouse); *P < 0.05, two-way ANOVA. (**D**) Lung microstructure visualized by L-IVM with reflected light (655 nm) shows alveoli and surrounding interstitial tissue, mainly containing microvessels. Scale bar, 50 μm. Alveoli appear as round areas bordered by the microvascular bed, highlighted by lines in (E) and (F). (**E** and **F**) L-IVM of control (E) and cQD-NP-exposed (16 cm^2^/g) mice (F) shows neutrophils (green; anti-Ly6G mAb) and alveolar structures (red; reflected light at 655 nm). Dotted lines mark alveolar boundaries [(E) control at −15 min (left) and 90 min (right); (F) cQD-NPs at −15 min (left) and 90 min (right)]. Scale bars, 20 μm. (**G**) Neutrophil infiltration into alveoli over time following cQD-NP (16 cm^2^/g) inhalation versus control. (**H**) Percentage of alveolar-localized neutrophils of the total number of neutrophils after cQD-NP (16 cm^2^/g) inhalation and under control conditions. Data are presented as means ± SEM in [(C), (G), and (H)]. *n* = 3 mice per group. *P < 0.05 and **P < 0.01, two-way ANOVA test. QD inhalation starts at 0 min (red arrow).

Close inspection of long-term L-IVM recordings of neutrophil dynamics upon cQD-NP inhalation suggested that neutrophils preferentially arrested in microvessels near the alveolar-deposited cQD-NPs, where they often exhibited a probing/crawling behavior (Fig. 2A). Therefore, the number of neutrophils close to deposited cQD-NPs was quantified in square areas (100 μm in side length) centered around cQD-NP “hotspots” (Fig. 2B) and compared to neutrophil counts in cQD-NP-deficient areas. The number of neutrophils increased significantly and more rapidly in close proximity to cQD-NP accumulations than in cQD-NP-deficient areas (Fig. 2C).

During L-IVM, the lung structure can be clearly imaged using reflected light microscopy; thus, the outlines of the alveoli are easily identifiable (Fig. 2, D to F). These imaging properties enable the discrimination of airspace and microvessels and, thus, also the differentiation between alveolar and microvessel-localized neutrophils (Fig. 2, E and F). Already 30 min after inhalation of cQD-NPs, an increased number of alveolar-localized neutrophils could be detected compared to control mice, and the increase became significant at 60 min (Fig. 2G), with the proportion of alveolar-localized neutrophils to total neutrophils increasing significantly faster than in the control animals over the course of time (Fig. 2H). Overall, these data suggest that the influx of neutrophils into the airspace starts within 30 to 45 min after inhalation of cQD-NPs.

Immunofluorescence (IF) staining of lung tissue sections of mice 90 min after cQD-NP inhalation frequently identified lung neutrophils with internalized cQD-NPs (fig. S2A), and in L-IVM images obtained 1 hour after cQD-NP inhalation, alveolar-localized neutrophils were associated with cQD-NPs, both indicating QDs uptake (fig. S2E). In agreement to that, analysis of BAL obtained 24 hours after cQD-NP inhalation showed that cQD-NPs were phagocytosed not only by macrophages (23.7 ± 1.2% of all BAL macrophages contain cQD-NPs) but also by neutrophils (15.6 ± 2.9% of all BAL neutrophils contain cQD-NPs) (fig. S2, B to D). Thus, these data indicate a contribution of neutrophils in the alveolar clearance of NPs.

### AMs exhibit enhanced patrolling and accumulate in NP-rich areas upon NP inhalation

**Fig. 3.**
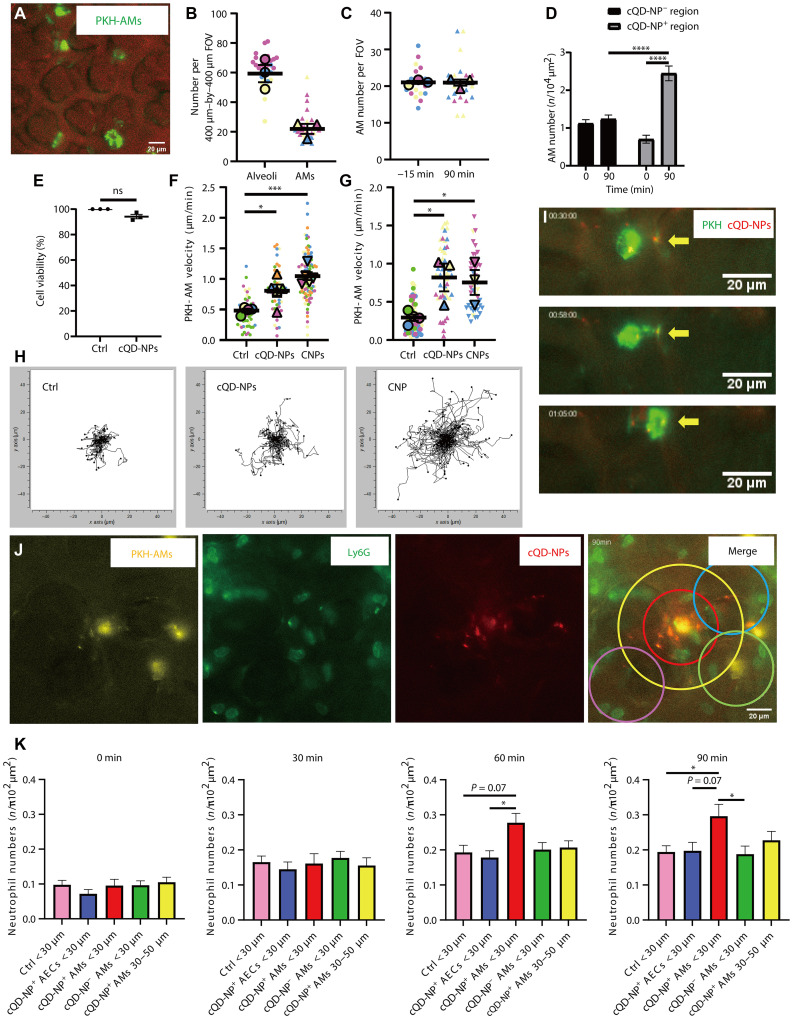
Alveolar deposition of NPs altered the patrolling speed of AMs and induced the recruitment of neutrophils that focally appeared around NP-containing AMs. (**A**) Representative L-IVM image showing PKH-labeled AMs (PKH-AMs) (green) and lung microstructure (reflected light; red). Scale bar, 20 μm. (**B**) Quantification of AM-to-alveolus ratios from L-IVM (7 to 14 random regions per mouse, *n* = 3 mice per group). Mean values per mouse are indicated by larger colored symbols. (**C**) PKH-AM counts 15 min before and 90 min after cQD-NP exposure (seven regions per mouse, *n* = 3 mice per group). Mean values per mouse are shown as larger colored symbols. (**D**) Increased AM numbers detected by L-IVM in cQD-NP-rich regions (100 μm in diameter) at 90 min postinhalation (*n* = 3 mice per group). (**E**) Viability of MH-S cells after 1 hour of cQD-NP exposure (8 nM; in vivo equivalent) assessed by water-soluble tetrazolium salt (WST) assay (*n* = 3). (**F** and **G**) Average AM track velocity (in micrometers per minute) during 1-hour L-IVM imaging at (F) 1 hour and (G) 24 hours post–NP exposure. Each symbol indicates one AM from four to six experiments (*n* = 4 to 6 mice per group); mouse means are shown as larger colored symbols. (**H**) Spider plots of PKH-AM migration tracks over 1-hour L-IVM in mice exposed to cQD-NPs or CNPs (10 to 20 AMs per mouse; *n* = 4 to 5 mice per group). (**I**) Time-lapse L-IVM of PKH-AM (green) migrating toward and engulfing cQD-NPs (yellow arrows). (**J**) L-IVM at 60 min post–cQD-NP inhalation: neutrophils (green) aggregate near PKH-AMs (yellow) with internalized cQD-NPs (red). Circles (radii, 30 and 50 μm) centered on cQD-NP^+^ PKH-AMs (red/yellow); also shown are cQD-NP^+^ PKH-AMs (blue), AECs (green), and control areas (pink). Scale bars, 20 μm. (**K**) Quantification of neutrophil accumulation in regions shown in (J) (*n* = 4). Data are presented as means ± SEM. ns, *P* ≥ 0.05; *P < 0.05, ***P < 0.001, and ****P < 0.0001.

AMs serve as main phagocytic cells in the alveolar lumen cavity, exhibiting robust capabilities in clearing inhaled pathogens and pollution-related particles (bacteria, viruses, and NPs), as well as cellular debris and lung surfactant, thereby maintaining lung homeostasis (*33*, *34*). To facilitate the investigation of AM motility and NP uptake and relate these to neutrophil responses, we labeled AMs by oropharyngeal aspiration of PKH26 dye (*26*). Our L-IVM images confirmed recent data from Neupane *et al.* (*26*) that not every alveolus contains an AM (Fig. 3A), with the ratio of AMs to alveoli being one to three (Fig. 3B). This again emphasizes the need for AMs to patrol within and between alveoli to maintain homeostasis of the alveolar microenvironment (*26*).

cQD-NP inhalation did not affect PKH-labeled AM (PKH-AM) cell numbers in alveoli in the analyzed field of views 15 min prior and 90 min post–cQD-NP inhalation, as determined by L-IVM (Fig. 3C). However, AMs accumulated over time close to deposited cQD-NP hotspots (Fig. 3D). Tracking of PKH-AMs 60 min postinhalation revealed that compared to control values, inhalation of cQD-NPs, as well as CNPs, increased the migration velocity of AMs in the alveoli of the respective mice (Fig. 3, F and H). Even 24 hours after cQD-NP and CNP exposure, AMs remained particularly active in the alveoli, exhibiting increased velocities compared to controls (Fig. 3G). Increased AM patrolling might thus facilitate effective NP clearance, as observed within the first hour post–cQD-NP inhalation, where AMs crawled toward and phagocytosed alveolar-deposited cQD-NPs (Fig. 3I). We do not anticipate cytotoxic effects of cQD-NPs, since incubation of a murine AM cell line, MH-S (*35*), with cQD-NPs did not affect cell viability (Fig. 3E).

### Neutrophil recruitment is initiated by cQD-NP^+^ AMs

Having observed that neutrophil recruitment occurs in the vicinity of NP “deposition hotspots” (Fig. 2C) (*36*–*38*), we next used spatial segmentation to analyze whether AMs or AECs contribute to initiating the local immune response at the alveolar level (mouse alveoli is ~30 μm) (Fig. 3J) (*39*). Therefore, neutrophil numbers in circular regions of interest (*r* < 30 μm) centered around (i) PKH26-labeled AMs colocalized with cQD-NPs (cQD-NP^+^ AM), (ii) AMs without cQD-NPs (cQD-NP^−^ AMs), (iii) cQD-NPs localized at/in alveolar walls/AECs, or (iv) control regions (cQD-NP and AM free) have been determined upon inhalation over time (Fig. 3K).

From 60 min after cQD-NP inhalation, significantly increased neutrophil aggregation appeared only around cQD-NP^+^ AMs compared to cell numbers detected adjacent to cQD-NP^−^ AMs and cQD-NP clusters localized at/in AECs and control regions (Fig. 3K). With increasing distance (from *r* < 30 μm to *r* = 30 to 50 μm), the tendency of neutrophils to gather in close proximity to the cQD-NP^+^ AMs decreased (Fig. 3K). Together, the data suggest that the cQD-NP-induced spatially restricted recruitment of neutrophils is initiated by particle-laden AMs rather than the adjacent AECs, with type I pneumocytes known to contribute to the surface of more than one alveolus.

### Inhibiting cellular degranulation diminishes NP-induced neutrophil recruitment

Considering that neutrophils accumulate close to NP-laden AMs and macrophages are known to release proinflammatory mediators upon stimulation of pattern recognition receptors and receptor-mediated phagocytosis, we set out to investigate whether inhibited cellular degranulation (by application of cromolyn) is similarly involved in pulmonary neutrophil recruitment as previously shown post–intravenous NP injection in skeletal muscle tissue (*28*). Noteworthy, cromolyn has been suggested to inhibit AM stimulation via plasma membrane stabilization (*40*).

**Fig. 4.**
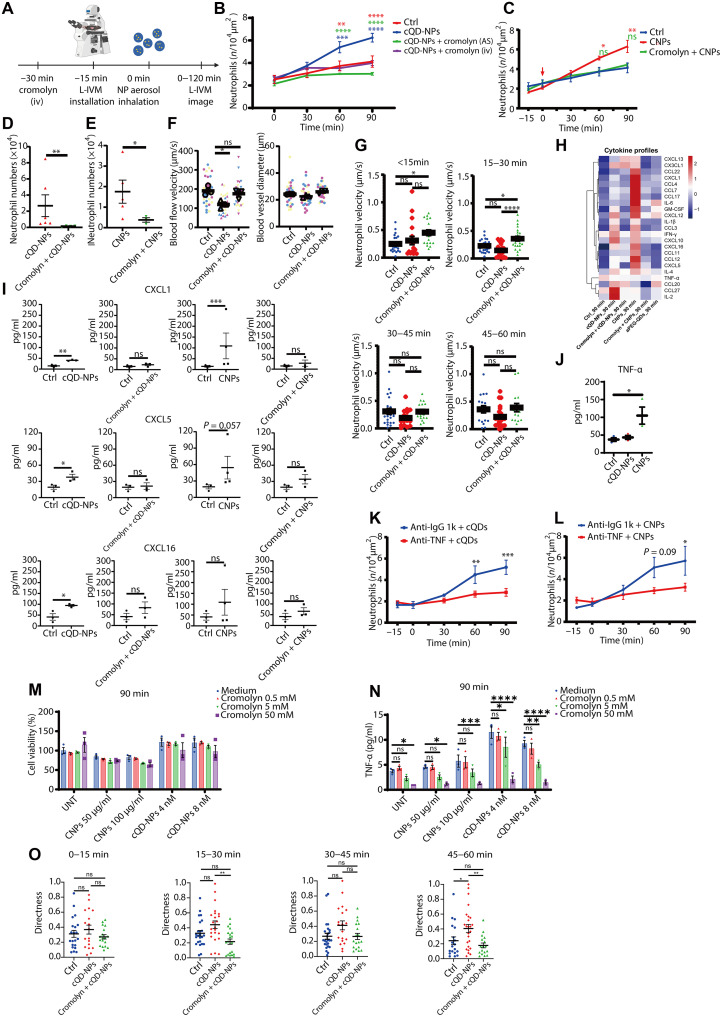
NP-induced neutrophil recruitment is initiated by cellular degranulation. (**A**) Schematic of cromolyn-mediated inhibition of cellular degranulation. (**B** and **C**) Neutrophil counts during L-IVM in mice pretreated with cromolyn [intravenously (iv) and/or oropharyngeal aspiration (AS); 0.2 mg/kg of BW] 30 min before NP inhalation of (B) cQD-NPs and (C) CNP or vehicle (*n* = 4 to 6 mice per group). (**D** and **E**) Neutrophil numbers in BAL 24 hours after inhalation of (D) cQD-NPs (*n* = 4) or (E) CNPs (*n* = 3), with or without cromolyn pretreatment. (**F**) Blood flow velocity in pulmonary microvessels 90 min after cQD-NP inhalation in mice ± cromolyn pretreatment (10 fluorescent beads per mouse; *n* = 3); vessel diameters are also shown (right). (**G**) Neutrophil crawling velocity with long residence time (>5 min) near cQD-NP-rich areas (<100 μm) after cQD-NP or vehicle inhalation ± cromolyn (six to seven cells per mouse, *n* = 3). (**H**) Heatmap of BAL cytokine/chemokine levels 90 min after NP inhalation [cQD-NPs, amine-PEG–QDs (aPEG-QDs), or CNPs] in naive or cromolyn-pretreated mice (*n* = 3 to 4). For each cytokine (each row in heatmap), values in different groups are centered and scaled in the row direction and visualized in a *z*-score distribution manner. GM-CSF, granulocyte-macrophage colony-stimulating factor; IFN-γ, interferon-γ. (**I**) Individual alterations of cytokines. (**J**) TNF-α levels in BAL measured by enzyme-linked immunosorbent assay (ELISA) 2 hours after inhalation of CNPs, cQD-NPs, or vehicle (*n* = 3). (**K** and **L**) L-IVM quantification of neutrophil dynamics after anti–TNF-α or isotype mAb pretreatment, followed by (K) cQD-NP or (L) CNP inhalation (*n* = 3). Inhalation begins at 0 min. (**M**) THP-1 macrophage viability after 90-min incubation with CNPs or cQD-NPs ± cromolyn (0.5, 5, and 50 mM) via WST-1 assay; no significant cytotoxicity was observed. UNT, untreated. (**N**) CNP- and cQD-NP-induced TNF-α release from THP-1 macrophages at 90 min is dose-dependently inhibited by cromolyn. (**O**) Directness of individual neutrophil migration trajectories under different treatment conditions. Data are presented as means ± SEM. ns, *P* ≥ 0.05; *P < 0.05, **P < 0.01, ***P < 0.001, and ****P < 0.0001, two-way ANOVA test.

Mice were pretreated by intravenous injection with cromolyn [0.2 mg/kg of body weight (BW)] 30 min before NP inhalation (Fig. 4A). Cromolyn pretreatment prevented cQD-NP- and CNP-induced neutrophil recruitment and neutrophil levels after NP inhalation matched those of the respective vehicle control group (Fig. 4, B and C). The anti-inflammatory effect of cromolyn was long lasting, and pretreatment with cromolyn prevented infiltration of neutrophils into the airspace for 24 hours post–NP exposure, as determined by BAL analysis (Fig. 4, D and E). Accordingly, cromolyn pretreatment restored blood flow velocity in cQD-NP-exposed mice to control levels with no discernible impact on pulmonary microvessel diameters (Fig. 4F). Direct application of cromolyn to the airways via aspiration also significantly reduced neutrophil recruitment into the alveolar space, thereby closely mirroring the effects of systemic treatment (Fig. 4B). The effects of cromolyn on neutrophil recruitment are therefore not limited to the systemic pathway and emphasize its role in modulating local inflammatory responses in the lung.

To further study the effect of cromolyn on cQD-NP-induced neutrophil recruitment, we analyzed the neutrophil crawling velocity along the microvessel walls. Following cQD-NP exposure, the speed of neutrophil movement along blood vessel walls near deposited cQD-NPs (from 15 min) was decreased compared with the situation in control mice, again demonstrating the rapidity of the neutrophil response. Pretreatment with cromolyn considerably increased microvascular neutrophil crawling velocities in cQD-NP-exposed mice, surpassing even the level of the control group (Fig. 4G), most pronounced in the 15- to 30-min period (Fig. 4G). This suggests that cellular degranulation slows down neutrophils in the proximity of deposited NPs, potentially intensifying crawling and probing behavior and subsequent leukocyte recruitment to the airspace in these areas.

To investigate whether cromolyn directly impacts the ability of macrophages to release cytokines, we assayed the release of tumor necrosis factor–α (TNF-α) after incubation with cQD-NPs and CNPs for 90 min, by THP-1 macrophages in vitro (Fig. 4N). In these experiments, a clear cromolyn concentration–dependent inhibition of TNF-α release (in a cromolyn concentration range reflecting in vivo conditions) was observed after NP exposure (CNPs at 50 or 100 μg/ml and cQD-NPs at 4 or 8 nM). TNF baseline secretion from THP-1 cells (possibly induced by differentiation) was also inhibited (Fig. 4N). Viability of the cells among the experimental groups was not affected by the different treatments indicating minimal cytotoxicity from NPs or cromolyn (Fig. 4M). Moreover, analysis of the directness of individual neutrophil migration trajectories [directness is used to characterize the straightness of migration, which is often related to chemotaxis (*41*), in which “1” represents a straight line between the start and end point] shows that cromolyn disrupts neutrophil responsiveness to local cues, presumably generated by AM activation and degranulation (Fig. 4O).

To characterize the release of proinflammatory mediators 90 min after inhalation of cQD-NPs and CNPs and assess the impact of cromolyn treatment, chemokine concentrations in BAL supernatants were quantified by use of a multiplex cytokine assay. Both cQD-NP and CNP inhalation resulted in a swift release of the neutrophil attractants chemokine ligand 3 (CCL3), C-X-C motif chemokine ligand 5 (CXCL5), and interleukin-1β (IL-1β). A rapid release of inflammatory factors was not observed in polyethylene glycol (PEG)–QD–exposed mice, which is also consistent with the lack of subsequent induction of neutrophilic inflammation after inhalation (Fig. 4H and the “NP phagocytosis by AMs is a key event in the initiation of rapid neutrophil recruitment” section). Furthermore, the rapid release of inflammatory factors could be effectively inhibited by application of the degranulation inhibitor, cromolyn (Fig. 4H). Even if local cytokine changes might be blurred upon BAL analysis, these results suggest that inflammatory factors, including the CXCL family (Fig. 4I), are involved in the rapid recruitment of neutrophils and might be released during cellular degranulation following exposure to NPs.

In addition, a focused examination of the BAL levels of the cytokine TNF-α after NP inhalation was conducted because of recent findings indicating its crucial role in neutrophil extravasation in skin inflammation by promoting intraluminal crawling (*42*). As shown in Fig. 4J, significantly increased TNF-α levels were detected 2 hours after CNP inhalation but not for cQD-NPs. Furthermore, TNF-α neutralization by oropharyngeal aspiration of anti–TNF-α monoclonal antibodies (mAbs) 3 hours before cQD-NP or CNP inhalation resulted in significantly reduced neutrophil accumulation over the observation period (Fig. 4, K and L), indicating that TNF-α signaling in the airspace is required for the rapid neutrophil recruitment upon NP inhalation. However, this response might be independent of a pronounced proinflammatory activation of AMs, since no evidence for an inflammatory activation of in vitro CNP-exposed macrophages could be observed after 3 and 9 hours, as assessed by the expression levels of classical proinflammatory genes and gene set enrichment analysis of hallmark signaling pathways (fig. S7). To test whether cromolyn can act directly on macrophages, we pretreated THP-1 macrophages with cromolyn and incubated them with CNPs and cQD-NPs for 90 min. Water-soluble tetrazolium salt 1 (WST-1) assay showed no cytotoxic effects of the respective treatments (Fig. 4M), whereas CNP- and cQD-NP-induced rapid TNF-α secretion was significantly inhibited by cromolyn in a dose-dependent manner (Fig. 4N). These findings strongly support the conclusion that AMs are a key source of proinflammatory mediators following NP exposure and initiate the innate immune response.

### AM patrolling ability being a prerequisite for NP clearance and subsequent neutrophil recruitment

**Fig. 5.**
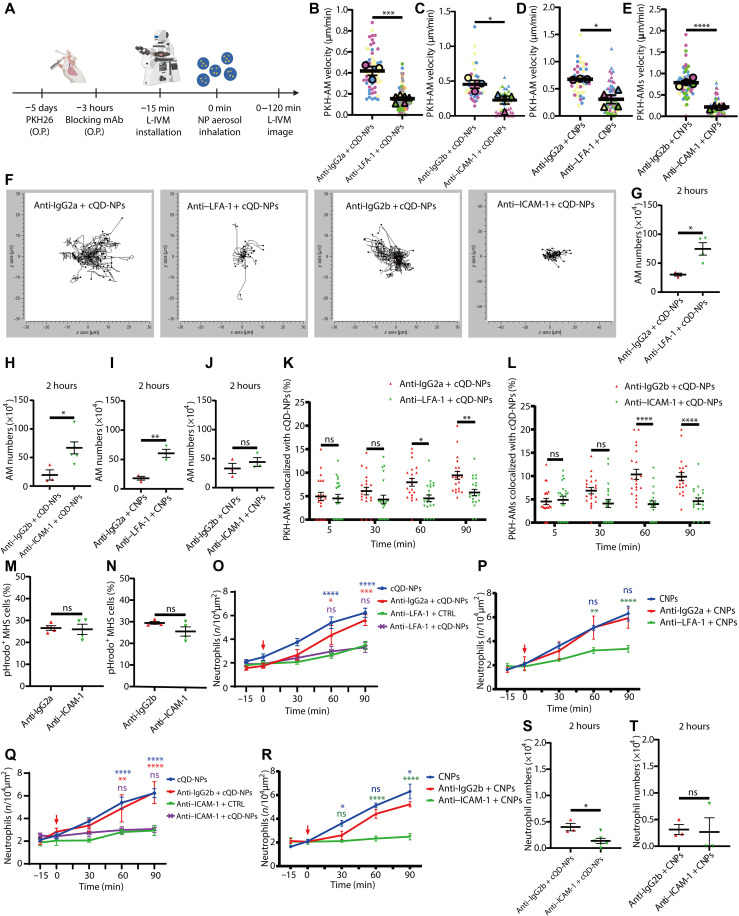
Inhibition of alveolar ICAM-1 and LFA-1 attenuates the uptake of NPs by AMs and the subsequent neutrophil recruitment. (**A**) Schematic of LFA-1 and ICAM-1 blockade, followed by NP inhalation in PKH26-labeled mice. (**B** to **E**) Average AM track velocity (in micrometers per minute) for 1 hour L-IVM in mice treated with isotype, anti–LFA-1, or anti–ICAM-1 mAbs, followed by cQD-NP or CNP inhalation (*n* = 3 to 6 per group). Larger symbols indicate individual mouse means. (**F**) Spider plots of representative AM migration paths in alveoli posttreatment as above (10 to 20 AMs per mouse; *n* = 3 to 6 per group). (**G** to **J**) AM counts in BAL fluid 2 hours after cQD-NP (G and H) or CNP (I and J) exposure in mAb-treated mice (*n* = 3 to 4 per group). (**K** and **L**) Quantification of AM-associated QDs in isotype versus anti–LFA-1 or anti–ICAM-1 mAb-treated mice post–cQD-NP inhalation (*n* = 3 to 4 per group). (**M** and **N**) Phagocytosis of fluorescent pHrodo *E. coli* particles (5 μg/ml) for 1 hour by MH-S cells pretreated with indicated mAbs, assessed via flow cytometry (*n* = 4 replicates). (**O** to **R**) Time course of neutrophil recruitment during L-IVM in mAb-pretreated mice after cQD-NP (O and Q) or vehicle control (CTRL) or CNP (P and R) exposure (*n* = 3 to 6 per group). (**S** and **T**) BAL neutrophil quantification 2 hours postinhalation of cQD-NPs (S) or CNPs (T) in isotype versus anti–ICAM-1-treated mice (*n* = 3 to 4 per group). Data are presented as means ± SEM. ns, *P* ≥ 0.05; *P < 0.05, **P < 0.01, ***P < 0.001, and ****P < 0.0001.

Next, we investigated whether AM patrolling and/or AM phagocytosis of NPs are critical for the spatially and temporally defined immune response. First, we addressed whether AM crawling, which was enhanced after NP inhalation (Fig. 3, F to H), is a prerequisite for the initiation of neutrophil recruitment. For this, we targeted the adhesion molecules CD11a/LFA-1 that are highly expressed on AMs and have been shown to be used by AMs to crawl on alveolar epithelium (*26*) and its epithelial expressed adhesion ligand ICAM-1 (intercellular adhesion molecule–1), which is also implicated in leukocyte migration into the lung (*42*–*44*). ICAM-1 and LFA-1 were neutralized in separate experiments by administration of the respective blocking antibodies, by oropharyngeal aspiration 3 hours before NP-inhalation into the lungs of mice (Fig. 5A). AM motility in anti–LFA-1 and anti–ICAM-1 mAb–treated mice was significantly decreased following cQD-NP and CNP inhalation, compared with isotype antibody–treated control mice (Fig. 5, B to F).

Following cQD-NP and CNP inhalation, more AMs could be recovered by lavage from anti–LFA-1 and anti–ICAM-1 antibody–treated mice compared to mice receiving isotype-matched control antibodies (Fig. 5, G to J). This implies that LFA-1/ICAM-1 blockade also impaired the ability of AMs to firmly anchor to the alveolar walls in vivo, leading to a significant reduction in the adhesion strengthening between AMs and lung epithelial cells, thereby further affecting the crawling and patrolling of AM on AECs.

Attenuated AM patrolling due to LFA-1/ICAM-1 blockade, affected the clearance of deposited NPs by AMs. Pretreatment via the airspace with anti–LFA-1 and anti–ICAM-1 mAbs before cQD-NP inhalation induced a significant reduction in cQD-NP internalization in AMs at 60 and 90 min as compared to isotype mAb–treated mice (Fig. 5, K and L).

AM phagocytosis capability was seemingly not altered by the application of anti–LFA-1 and anti–ICAM-1 antibodies, since MH-S cells incubated with anti–LFA-1 or anti–ICAM-1 antibodies for 3 hours displayed no significant difference in phagocytosis of pHrodo *Escherichia coli* particles (*45*) at 60 min compared with isotype-treated MH-S cells (Fig. 5, M and N).

Together, this suggests that the lower efficiency of NP phagocytosis by AMs in vivo upon blocking LFA-1/ICAM-1 is due to the mitigating effect on AM motility rather than on AM phagocytosis. This supports the view that AMs rely on ICAM-1/LFA-1 to migrate on the alveolar epithelium to clear deposited NPs in the alveoli.

In addition, prior application of anti–LFA-1, as well as anti–ICAM-1 mAbs, by aspiration to the airspace, diminished neutrophil recruitment induced by cQD-NP and CNP inhalation effectively, as compared to the respective isotype mAb and vehicle controls at 60 and 90 min in L-IVM experiments (Fig. 5, O to R). This was partially confirmed by quantification of neutrophils in the BAL fluid of these mice at 2 hours (Fig. 5, S and T).

To rule out that the diminished neutrophil recruitment is caused by the diffusion of antibodies across the epithelial barrier and subsequent direct inhibitory interaction with endothelial cells and neutrophils, we administered LFA-1 and ICAM-1 blocking antibodies in separate experiments, via intravenous injection, directly into the circulatory system at concentrations, which blocked TNF-α–induced (for LFA-1) (*46*) or cQD-NP-induced (for ICAM-1) (*28*) leukocyte recruitment in skeletal muscle tissue. Intravenous application of anti–ICAM-1 blocking mAb in mice receiving cQD-NPs by inhalation elevated the numbers of recruited neutrophils in the lungs, compared to wild-type (WT) mice receiving cQD-NPs, whereas anti–LFA-1 mAbs did not substantially attenuate cQD-NP-induced neutrophil recruitment (fig. S3, A and B). Together, the above results support that blocking epithelial ICAM-1 and AM LFA-1 results in the inhibition of cQD-NP- and CNP-induced neutrophil recruitment and provide insights into their roles in the processes taking place in the intravascular compartment.

### NP phagocytosis by AMs is a key event in the initiation of rapid neutrophil recruitment

**Fig. 6.**
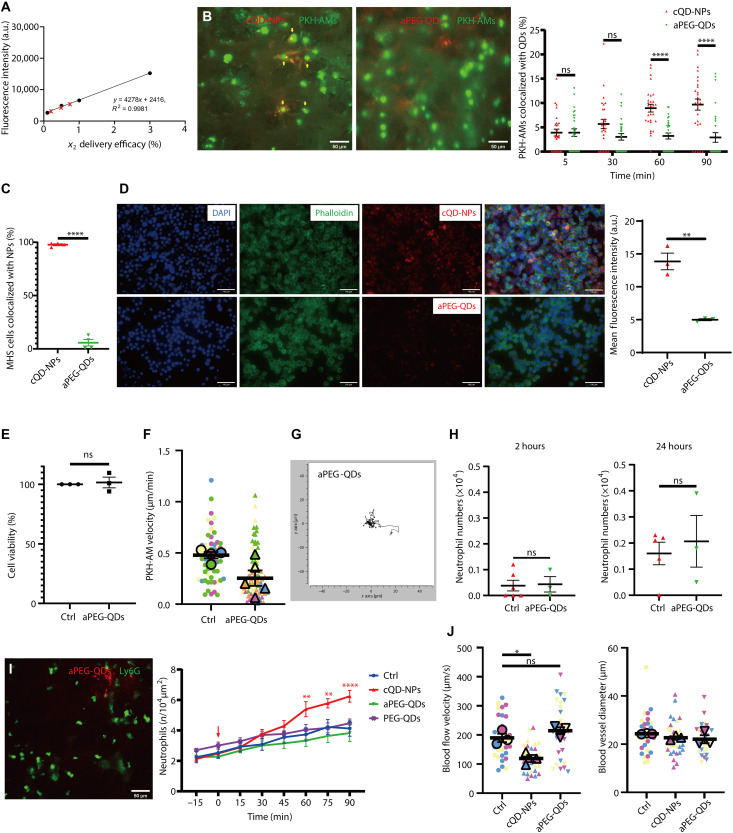
PEGylation reduces NP-induced neutrophil recruitment due to preventing the recognition and internalization of NPs by AMs. (**A**) Quantification of lung-deposited aPEG-QDs postinhalation, based on a linear fluorescence standard curve (*R*^2^ > 0.99). Red crosses indicate detected lung levels (0.51 ± 0.17% of input dose, means ± SEM; *n* = 3, three independent experiments), consistent with cQD-NP deposition efficiency (16 cm^2^/g) (Fig. 1E). (**B**) L-IVM images (90 min) show QD (red) uptake by PKH-AMs (green); yellow indicates colocalization. Scale bars, 50 μm. Right: Quantification of AM-QD colocalization across seven FOVs per time point per mouse (*n* = 3 per group). (**C**) In vitro uptake of cQD-NPs and aPEG-QDs by MH-S cells quantified via FACS after 1-hour incubation (*n* = 4). (**D**) Representative epifluorescence images of MH-S cells [phalloidin, green; 4′,6-diamidino-2-phenylindole (DAPI), blue] grown at air-liquid interface (ALI) 2 hours postexposure to cQD-NPs or aPEG-QDs (red) with the VITROCELL cloud system. Scale bars, 100 μm. Right: Quantification of QD fluorescence (FOV, 680 μm by 512 μm; *n* = 3). (**E**) Cell viability of MH-S cells after 1-hour exposure to aPEG-QDs (8 nM), assessed by WST assay. (**F**) AM track velocity (in micrometers per minute) during 1-hour L-IVM in NP-exposed mice (symbols, individual AMs; larger markers, mouse means; *n* = 4 to 5). (**G**) Spider plots of AM migration in alveoli post–aPEG-QD exposure (10 to 20 AMs per mouse; *n* = 4 to 5). (**H**) BAL neutrophil counts at 2 and 24 hours post–aPEG-QD or vehicle inhalation (*n* = 3 to 6 per group). (**I**) Left: L-IVM images at 60 min postinhalation of aPEG- or PEG-QDs (red) and neutrophils (anti-Ly6G; green). Scale bar, 50 μm. Right: Quantification of neutrophils after cQD-NP, aPEG-QD, PEG-QD, or vehicle exposure (deposited dose, 16 cm^2^/g; *n* = 6 per group). (**J**) Left: No change in alveolar microvessel diameter across treatments. Right: Blood flow velocity at 90 min postinhalation, measured via fluorescent bead tracking (larger markers, mouse means). Data are presented as means± SEM for *n* = 3 mice per group. ns, *P* ≥ 0.05; *P < 0.05, **P < 0.01, and ****P < 0.0001.

“Stealth” QDs that are not readily recognized for phagocytosis by macrophages were applied to further characterize the role of NP phagocytosis by AMs in the initiation of neutrophil recruitment. Their stealth feature is due to the modification/coating of the QD surface with PEG, which largely reduces biomolecule binding (*28*, *47*–*49*). For this purpose, amino-PEG–QDs (aPEG-QDs) of the same size were used, which show identical deposition efficiency (0.51 ± 0.17%; Fig. 6A) and dose as cQD-NPs in the lungs after aerosolization and inhalation (deposited dose determined by quantitative fluorescence, 16 cm^2^/g; Fig. 6A).

Low levels of AMs colocalized with QDs at 5 min after inhalation (median < 3%), and the percentage of colocalization is gradually increasing for cQD-NPs over the observation time of 90 min but not for aPEG-QDs as detected by L-IVM (Fig. 6B). To investigate this further, MacGreen mice were used, where colony stimulating factor 1 receptor (Csf1r)–enhanced green fluorescent protein (EGFP) is expressed selectively in macrophage and monocyte cell lineages. Accordingly, higher numbers of QD-positive AMs were observed in precision cut lung slices obtained from MacGreen mouse lungs having inhaled cQD-NPs; this is not seen for aPEG-QDs (fig. S4, A and B).

To verify QD-dependent uptake by AMs in vitro, we exposed AM-like MH-S cells, cultivated at the air-liquid interface (ALI) (*50*), to aerosolized cQD-NPs or aPEG-QDs for 1 hour. Qualitative analysis was performed by confocal microscopy on MH-S cell cultures exposed via the ALI system (Fig. 6C). For quantitative analysis, we conducted flow cytometry [fluorescence-activated cell sorting (FACS)] on cells exposed in medium, confirming that 95.8 ± 1.8% of MH-S cells internalized cQD-NPs, whereas only 8.8 ± 1.5% phagocytosed aPEG-QDs (Fig. 6D and fig. S8A). Cell viability of MH-S cells was not reduced by aPEG-QDs, i.e., the low uptake of aPEG-QDs is not due to cytotoxicity (Fig. 6E). Returning to in vivo conditions, L-IVM revealed that the average AM patrolling velocity increases in the presence of cQD-NPs relative to the negative control group, while it decreases after inhalation of aPEG-QDs (Fig. 6, F and G).

To substantiate AM phagocytosis as the key event for NP inhalation triggered neutrophil recruitment, we additionally used pure PEG-QDs, without amine surface functionalization. All three types of QDs exhibit the same core structure, display identical diameter (20 nm) following nebulization from suspensions (fig. S5, A to C), and appear uniformly distributed through the whole lungs after inhalation (fig. S5, D to F), with comparable lung delivery efficiency, as assessed by fluorescence imaging in excised lungs (Figs. 1E and 6A). Merged images of maximum intensity projections of QD fluorescence signals with the three-dimensional (3D) lung structure confirm that cQD-NP and aPEG-QDs exhibited a uniform distribution in the lungs in the overall view (fig. S5, J and K). However, some local differences existed at the microscopic scale. After cQD-NP exposure, preferential central acini (blue arrow) exhibited strong cQD-NP fluorescence, while in the great majority of the peripheral acini (yellow arrow), fewer cQD-NPs were deposited (fig. S5, J and L). Conversely, after aPEG-QD inhalation, the distribution of aPEG-QDs was similar in central or peripheral areas of the lungs (fig. S5, K and M). In summary, cQD-NPs exhibited larger aggregate sizes (“dot-like” pattern) and more heterogeneous, patchy, and more central deposition pattern than the more stable suspended aPEG-QDs. L-IVM revealed that inhalation of cQD-NPs and PEG-QDs resulted in a roughly twofold lower average fluorescence intensity in the respective fields of view than aPEG-QD inhalation (fig. S5, G to I and N).

In contrast to cQD-NPs, inhalation of aPEG-QDs and PEG-QDs did not result in rapid neutrophil recruitment detected by L-IVM relative to vehicle control after QD inhalation (Fig. 6I). This lack of aPEG-QD–induced neutrophil recruitment is corroborated by unchanged neutrophil numbers in BAL at 2 and 24 hours after aPEG-QD inhalation (Fig. 6H). Because of the lack of an inflammatory response to these stealth QDs, blood flow velocities also remained unchanged after inhalation of both aPEG-QDs and PEG-QDs (Fig. 6J).

To investigate cellular uptake and subcellular distribution of NPs, we applied transmission electron microscopy (TEM) to the lungs of mice after exposure to cQD-NPs, aPEG-QDs, or PEG-QDs for 1 hour. Some cQD-NP clusters were identified within the cytoplasm or in endolysosomes of AMs, whereas no such aggregated QDs could be identified in AMs in the PEG-QD or aPEG-QD groups (fig. S6A). cQD-NPs were found to be attached to or agglomerated with alveolar surfactants (tubular myelin sheets, based on their ultrastructure) at the air-liquid epithelial interface, which was not observed for aPEG-QD– and PEG-QD–exposed mice (fig. S6B). The TEM results confirm the lack of AM uptake for aPEG-QDs and PEG-QDs and thus support the stringent necessity of NP phagocytosis by AMs for the initiation of the inflammatory response.

### C5a receptor 1 and Fc-γ receptor I functions are crucial for rapid neutrophil recruitment upon NP exposure

**Fig. 7.**
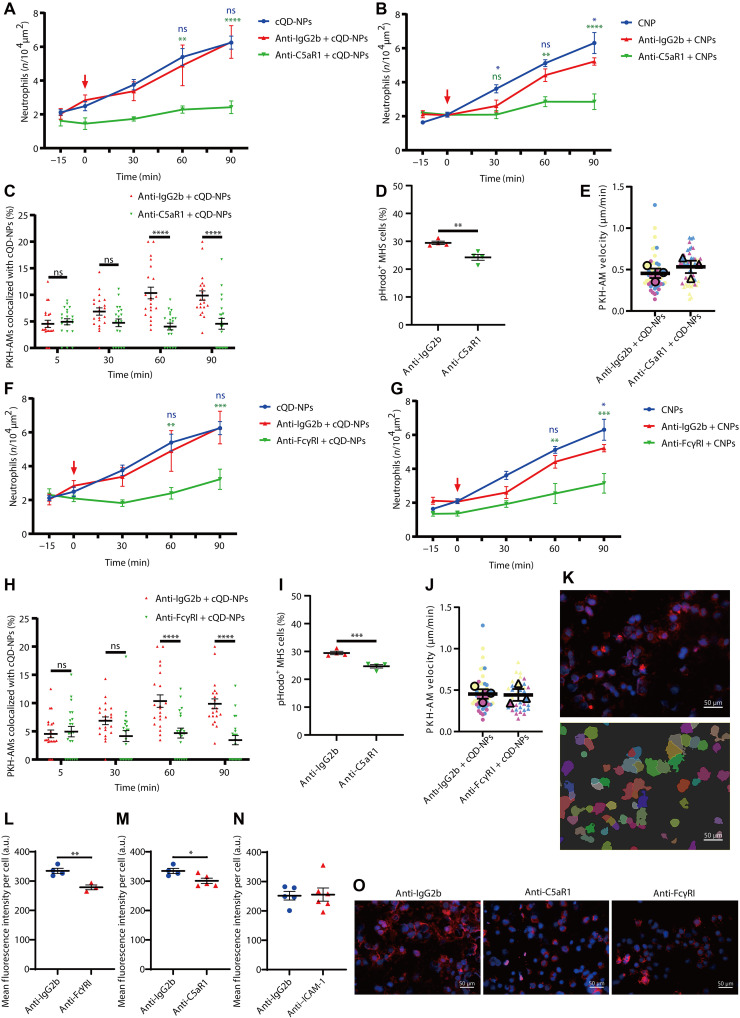
AM FcγRI and C5aR1 are involved in the rapid neutrophil recruitment upon NP exposure. (**A** and **B**) Neutrophil dynamics over time (L-IVM) in mice pretreated with anti-C5aR1 or isotype mAb before inhalation of cQD-NPs (A), CNPs (B), or vehicle (*n* = 3 to 6 per group). (**C**) Quantification of cQD-NP uptake by AMs in anti-C5aR1 or isotype mAb–pretreated mice, 3 hours before cQD-NP inhalation (*n* = 3 per group). (**D**) Phagocytosis of pHrodo *E. coli* by MH-S cells pretreated with anti-C5aR1 or isotype mAb, analyzed by flow cytometry after 1 hour incubation (*n* = 4 per group). (**E**) AM track velocity (in micrometers per minute) during 1-hour L-IVM following cQD-NP inhalation in anti-C5aR1 or isotype mAb–treated mice. Individual means are shown (*n* = 3 per group). (**F** and **G**) Neutrophil dynamics (L-IVM) in mice pretreated with anti-FcγRI or isotype mAb before cQD-NP (F), CNP (G), or vehicle inhalation (*n* = 3 to 6 per group). (**H**) cQD-NP uptake by AMs in anti-FcγRI or isotype mAb–treated mice, 3 hours before inhalation (*n* = 3 per group). (**I**) In vitro phagocytosis of pHrodo *E. coli* by MH-S cells pretreated with anti-FcγRI or isotype mAb, analyzed after 1 hour (*n* = 4 per group). (**J**) AM track velocity (in micrometers per minute) during 1-hour L-IVM following cQD-NP inhalation in anti-FcγRI or isotype mAb–treated mice. Individual means are shown (*n* = 3 per group). (**K**) cQD-NP fluorescence in MH-S macrophages analyzed by Imaris cell module segmentation. (**L** to **N**) Mean QD fluorescence per cell in MH-S macrophages pretreated with anti-FcγRI (L), anti-C5aR1 (M), or anti-ICAM-1 (N) or isotype control, followed by QD incubation. (**O**) Representative image of MH-S macrophages showing QD signal (red) and nuclei (blue) (*n* = 3 to 5). Scale bars, 50 μm. Data are presented as means ± SEM. ns, *P* ≥ 0.05; *P < 0.05, **P < 0.01, ***P < 0.001, and ****P < 0.0001.

Since NP phagocytosis was identified as a key event, we were interested in the involved uptake receptors. The complement receptor C5a receptor 1 (C5aR1) (CD88) is highly expressed on AMs (Fig. 7A) (*26*, *51*) and thus served as a good candidate. AMs are known to get rapidly stimulated by C5a to chemotaxis and uptake of bacteria (*26*), and C5a is closely related to cellular degranulation (*52*, *53*). To assess whether the C5a-C5aR1 axis was vital in neutrophil recruitment after NP inhalation, we applied C5aR1-blocking (CD88) mAbs by oropharyngeal aspiration, followed by cQD-NP and CNP aerosol inhalation. The application of anti-C5aR1 mAbs effectively prevented the rapid neutrophil recruitment after cQD-NP inhalation (Fig. 7A), as well as after CNP inhalation (Fig. 7B). Likewise, only baseline levels of cQD-NP uptake by AMs were detected in the intravital images of anti-C5aR1–treated mice as compared to isotype mAb–pretreated mice (Fig. 7C), and, upon cQD-NP exposure, AM velocity was not affected by anti-C5aR1 mAbs pretreatment (Fig. 7E). Investigating pathogen phagocytosis using pHrodo *E. coli* particles exposed to MH-S cells for 3 hours with anti-C5aR1 mAbs under in vitro conditions showed significant decreased uptake at 60 min (Fig. 7D and fig. S8B), and the uptake of cQD-NPs was also significantly reduced in MH-S macrophages (Fig. 7, M and O), with cells segmented automatically using the cell module in Imaris software (Fig. 7K).

Together, these results suggest that blocking C5aR prevents NP-induced neutrophil recruitment due to reduced phagocytic ability/recognition of NPs by AMs without affecting AM patrolling behavior. Similar to F4/80 or the protein tyrosine kinase MER, Fc-γ receptor I (FcγRI) is specifically expressed on the surface of mouse macrophages (*54*) (fig. S7A). On the basis of earlier studies in primary murine macrophages, FcγR knockout mouse models, and human monocyte-derived macrophages, FcγR-mediated phagocytosis was more efficient at inducing inflammation than complement receptor-mediated phagocytosis (*55*, *56*). Since FcγR is also closely involved in the degranulation of many kinds of inflammatory cells, such as natural killer (NK) cells and mast cells (*57*–*60*), it presented another candidate uptake receptor to be investigated. To determine FcγRI function on NP-induced neutrophil recruitment, we blocked FcγRI with anti-CD64 mAbs administered into the lungs 3 hours before NP inhalation. Sixty minutes after cQD-NP or CNP exposure, neutrophil accumulation in the lungs of anti-CD64–treated group was reduced compared to the isotype control group (Fig. 7, F and G). After anti-FcγRI mAb treatment, NP phagocytosis of AMs was again greatly reduced compared to isotype mAb–treated mice (Fig. 7H), while it had no effect on the patrolling movement of AMs (Fig. 7J). Again, pHrodo *E. coli* phagocytosis by MH-S cells was significantly attenuated by incubation with FcγRI blocking mAbs, in comparison to the isotype-treated group (Fig. 7I and fig. S8B), consistent with the findings for cQD-NPs (Fig. 7, L and O). Treatment with anti–ICAM-1 appears to influence the motility of AMs without affecting their phagocytic capacity (Fig. 7N). Together, these results support NP phagocytosis by AMs as a crucial event in the NP-induced alveolar neutrophil recruitment cascade, presumably followed by the release of proinflammatory mediators by AMs.

## DISCUSSION

Exposure to NPs, especially through ultrafine PM, a crucial component of ambient air pollution, is unavoidable in everyday life and is associated with notable health risks, not only concerning the development of cardiovascular diseases (*61*) but also in terms of acute and chronic respiratory symptoms and diseases (*62*–*64*). Nano-sized particles specifically have been shown to be of great concern, as they effectively reach the alveolar space and have a larger mass-specific surface area, which is one of the main drivers of particle-induced lung inflammation (*65*). In addition to a possible translocation of nanosized particles, the immediate inflammatory reaction in the lungs is critical for cardiovascular consequences (*8*, *66*). However, the initiation of complex innate immune reactions, which is triggered by NPs in humans and mice, remained largely unresolved so far. While it is widely accepted that particle surface interactions with biological membranes trigger oxidative stress (*67*), the specific cellular players responsible for triggering neutrophil recruitment at NP deposition sites are still unclear. Prime candidates are tissue-resident AMs, previously called “dust cells,” which, as resident phagocytes of the alveoli, play an essential role in the homeostatic “vacuum cleaning” of pulmonary surfactant turnover, daily cellular debris (*68*). The use of pHrodo particles, a pH-sensitive dye that is fluorescent only in the acidic phagosomal environment, provided functional evidence for internalization of NPs by AM. Specifically, colocalization observed in high-resolution imaging (e.g., L-IVM) indicates that NPs and AMs are in spatial proximity, while pHrodo fluorescence confirms active engulfment and internalization into acidic regions, distinguishing true phagocytosis from simple surface attachment. Together, these approaches—visualized colocalization and functional pH-dependent signaling—strongly validate NP uptake by AM in both in vivo and in vitro settings. Consistent with our current observations, efficient phagocytosis of NPs by AM was documented in previous publications of our group (*7*, *69*), where imaging and flow cytometry showed high rates of NP uptake by AMs.

AMs are essential for pathogen elimination via nonspecific phagocytosis and are strategically placed to initiate a robust inflammatory response to more pathogenic stimuli (*18*). Accordingly, AMs are also involved in the clearance of inhaled nanomaterials (*9*), but their role in the initiation of inflammation upon pulmonary deposition of sterile, pyrogen-free particles is controversial. Depending on the kind and cytotoxicity of inhaled PM, AMs have been considered to become activated upon contact or uptake and to produce proinflammatory cytokines or even undergo various cell death pathways (*70*, *71*). However, these findings are usually based on in vitro studies obtained using ambient PM samples containing various amounts of endotoxin or very high doses of highly toxic materials [e.g., residual oil fly ash of medium (100 μg/ml), i.e., nominal cellular dose of cells (0.3 μg/cm^2^) corresponding to 114 years of realistic urban particle exposure at 10 μg/m^3^] (*72*–*74*). In contrast to the common assumption of a proinflammatory AM activation by inhaled soot-like NPs, our earlier in vivo study showed at least no transcriptional activation of AMs in the lung of mice 6 and 12 hours after intratracheal instillation of 20 μg of CNPs (*7*). In addition, profiling murine bone marrow–derived macrophages, as shown in fig. S7, which are known to be prone to proinflammatory stimulation in contrast to AMs, revealed no inflammatory signature at the transcriptional level upon dosing with CNPs. Accordingly, macrophages respond differently to interaction with sterile particles or pathogens were the latter causes a robust stimulation, as indicated by endotoxin exposure (*75*).

The accumulation of neutrophils as the first responders of the innate immune system is considered the hallmark of inflammation (*21*). Typically, endotoxin (LPS) application to the lung is used as a positive control to benchmark neutrophil recruitment, offering a well-established model of acute pulmonary inflammation. In line with this, our prior work demonstrated that NPs could elicit similar inflammatory responses through endogenous danger signals such as extracellular adenosine 5′-triphosphate, a classic pathogen-associated molecular pattern–like LPS application (*32*). These observations suggest that the pathways targeted by cromolyn, anti–LFA-1, and anti–ICAM-1 extend beyond NP exposure and likely underlie core mechanisms of both sterile and pathogen-driven lung inflammation. In addition, upon NP exposure, neutrophils are rapidly recruited to the airspace, as described in various rodent models (*7*, *76*). Both AMs and neutrophils are sentinel cells that patrol, explore, and eliminate threats within the host (*21*, *26*). To date, the functions of these two types of immune cells during short-term exposure to inhaled NPs remain poorly understood, and their interplay in the response to alveolar-deposited NPs has not been investigated in their natural environment, i.e., the breathing lung. Combining L-IVM and ventilator-assisted NP inhalation enabled us to study dose-aware NP deposition, AM motility, and cellular NP uptake in the living organism and therefore to identify key events leading to spatiotemporally resolved neutrophil recruitment induced by inhaled NPs.

Using the PKH26L labeling method for resident phagocytes implemented by the Kubes laboratory to visualize AMs, our study confirmed their finding that, on average, one AM maintains the homeostasis of three murine alveoli (*26*). AMs exhibited crawling movements necessary to clean the alveolar surface from aged and excessive surfactant, cell debris, and NPs. In our L-IVM study, inhalation of both cQD-NP and CNP at equivalent inflammogenic dose levels almost doubled the AM migration velocity from around 0.5 to 1 μm/min, an effect not observed for PEG-shielded aPEG-QDs. Notably, while AM migration patterns in the alveoli appeared to be randomly oriented, directed movement toward NP clusters was observed over short distances (~10 to 20 μm). The observed net increase in AM numbers in the close vicinity of deposited NP hotspots might have been facilitated by an accelerated random walk. The change in AM migration thereby represents the first cellular event, initiating the pulmonary inflammatory response, as spatial mapping clearly indicated that NP-internalizing AMs prompted neutrophils to preferentially arrest in nearby microvessels.

The crucial role of AMs in pulmonary inflammation has been well documented, particularly in models of fiber-induced inflammation by experimental depletion of AMs, which decreased carbon nanotube–induced neutrophilic inflammation (*77*). In our hands, using established protocols for clodronate liposome–mediated AM depletion resulted in elevated neutrophil numbers in the airspace, higher than those induced by the low-dose NP inhalation challenge used here, rendering this approach, at least for our sensitive experimental approach, useless. In addition, recent critiques of clodronate liposome methodologies raise concerns about the interpretation of these studies (*78*). Consequently, we recognize the absence of AM-specific depletion data in this study as a limitation. However, application of for example the CD169-diphtheria toxin receptor model, which enables diphtheria toxin–dependent depletion of CD169^+^ cells (primarily resident AMs) with minimal induction of off-target inflammation (*79*), was beyond the scope of this work. Effective NP clearance by AMs hinges on both their motility and phagocytic capabilities. Our data indicated that AMs react to the alveolar presence of NPs (CNP and cQD-NPs), with increased motility, which turned out to be dependent on the expression of ICAM-1, the adhesion ligand for the β_2_ integrin LFA-1. ICAM-1 is highly expressed by murine type 1 pneumocytes and induced in inflammatory lung disease (*80*). LFA-1 (*Itgal*) gene knockout weakened AMs motility and impaired clearance of endogenous cellular debris in the alveolar space (*26*). In our study, both anti–LFA-1 and anti–ICAM-1 pretreatment alleviated the cQD-NP-induced increase in AM patrolling speed. LFA-1 or ICAM-1 blockade did not affect the phagocytic ability of AMs in in vitro experiments. Therefore, the inhibition of LFA-1 and ICAM-1 is expected to reduce clearance of cQD-NPs by AMs due to reduced AM motility in the alveoli but not due to mitigated AM phagocytic capacity. AM-epithelial interactions were also required to trigger early neutrophil attraction, as LFA-1 and ICAM-1 blocking antibody application eliminated the rapid recruitment of neutrophils occurring within the first hour after NP inhalation. As described by Neupane *et al.* (*26*), global LFA-1 knockout mice exhibit impaired AM displacement, demonstrating that LFA-1–dependent crawling is critical for alveolar homeostasis. Notably, intratracheal anti–LFA-1, which does not cross the epithelial barrier, selectively slowed AM motility without affecting vascular leukocytes. In agreement, we found that airway-delivered anti–LFA-1 reduces AM patrolling speed in vivo. By contrast, systemic (intravascular) LFA-1 blockade did not diminish cQD-NP-induced neutrophil influx, indicating that altered AM behavior, not impaired neutrophil adhesion, underlies the phenotype. To definitively dissect AM versus neutrophil contributions, future studies should use *Itgal*^flox/flox^ mice crossed with AM-specific (e.g., *Siglec F*–Cre or *Csf1r*-Cre) and neutrophil-specific (e.g., *Ly6G*-Cre) drivers for cell type–restricted deletion of LFA-1. The alveolar surfactant layer plays a pivotal role in preventing alveolar collapse by reducing surface tension. With its protein-rich lipids, lung surfactant can generate a biocorona wrapping alveolar-deposited NPs, subsequently modulating NP-cell interactions (*81*, *82*). Our TEM images showed the association of cQD-NPs with alveolar surfactant structures, thereby highlighting the high affinity of cQD-NP-NPs for biomolecular interactions. AMs have various surface receptors for pathogen identification and internalization (*83*, *84*), with particle uptake potentially facilitated by opsonization via complement factors or surfactant proteins. Pathogens are known to effectively induce macrophage and AM activation via pattern recognition receptors, particularly TLR2 and TLR4 for bacteria; however, these receptors are not required for NP-related neutrophilia (*20*). In this study, targeting FcγRI and C5aR1, both highly expressed on the surface of AMs but not on lung epithelial cells, eliminated NP-induced neutrophil recruitment without altering the patrolling status of AMs and resulted in defective NP recognition/phagocytosis by AMs. Surface PEGylation forms a steric barrier and reduces the opsonization of NPs (*47*), allowing PEGylated QDs to evade phagocyte recognition (*28*). The uptake of inhaled PEGylated QDs by AMs and the subsequent recruitment of neutrophils was significantly reduced, indicating that biological interactions with the surface area of pulmonary-deposited NPs is required for acute NP lung inflammation (*5*) and for NP availability for AM uptake.

Various immune cells including NK cells, eosinophils, and mast cells store inflammatory mediators in intracellular vesicles known as granules (*85*). These vesicles play a crucial role in releasing mediators during the host immune response to ensure immediate cell communication and defense. Previously, our group demonstrated that preventing cellular degranulation with cromolyn completely eliminated cQD-NP-evoked leukocyte recruitment in skeletal muscle postcapillary venules (*28*). Consistent with these findings, Lê *et al.* (*86*) revealed that cromolyn pretreatment greatly reduced TNF-α levels and lung myeloperoxidase activity in LPS-treated mice (*86*, *87*). A direct involvement of alveolar-localized mast cells in local neutrophil recruitment seems actually less likely, since mast cells are predominantly located in the central airways rather than peripheral alveoli of mice (*87*), which is in accordance with our own results, in which we could not detect mast cells in the alveolar regions, where L-IVM analysis was conducted (*32*). Moreover, the presence of mast cells in the lung parenchyma has recently been linked to the hygiene status of the mice. Mice housed under “specific pathogen–free” conditions are almost devoid of these cells, in contrast to wild mice (*88*). In our study, inhibiting degranulation by cromolyn led to a marked reduction in cQD-NP- and CNP-induced cytokine levels in the BAL to control levels (e.g., CXCL10, CXCL12, CCL3, and IL-1β) and prevented neutrophil recruitment up to 24 hours after NP inhalation; furthermore, we demonstrated in vitro that cromolyn directly acts on macrophages decreasing TNF-α secretion. These results might provide a previously unknown direction for mitigating the harm of NP exposure in the future. Furthermore, our data revealed that AMs served as critical effector cells in NP-induced neutrophil recruitment, a key event also requiring rapid TNF-α release. Since AMs are apparently not getting transcriptionally activated by NP uptake (*7*, *75*), the rapid release of inflammatory mediators through degranulation might represent a more immediate and temporal and spatial restricted mechanism for initiating a local immune response. The role of AMs in driving both acute and long-lasting inflammatory responses, after exposure to differently shaped NPs, has been further addressed through single-cell transcriptomics by our group (*89*).

Our results indicate that cromolyn attenuates neutrophil recruitment at least partly by inhibiting AM-derived proinflammatory cytokines, particularly members of the CXCL family. However, we acknowledge that cromolyn can also directly impair neutrophil chemotaxis and activation (*90*, *91*), which may further contribute to the observed reduction in neutrophil influx. The absence of detectable transcriptional activation in response to CNP exposure, despite robust early cytokine release both in vivo and in vitro, indicates that this acute inflammatory response does not depend on de novo gene expression. Rather, our data support a model in which NPs trigger the rapid release of preformed inflammatory mediators (e.g., TNF-α and CXCL chemokines) via a degranulation-like mechanism in AMs. This posttranscriptional pathway enables an immediate macrophage response, allowing for swift neutrophil recruitment before transcriptional programs can be engaged. By highlighting the reliance on stored mediator release, our findings underscore the need to consider nongenomic regulatory mechanisms, such as degranulation and vesicular exocytosis, when dissecting early lung immune responses to sterile particulates. Future work should explore the molecular machinery underpinning this rapid mediator discharge. Notably, there is limited research on neutrophils scavenging inhaled NPs, with most studies focusing on their role in clearing biological pathogens in lung microbe-infection models (*26*, *92*–*94*). Recently, the function of neutrophils in NP clearance from the bloodstream, before being diverted to the liver, has been highlighted (*95*). While the mononuclear phagocytic system primarily handles NP elimination from the bloodstream (*96*, *97*), our observations indicate that neutrophils play a role in NP clearance from the alveolar space. With L-IVM, we identified a substantial NP (cQD-NP) fraction, internalized by alveolar-localized neutrophils, recoverable in BAL. To which extent particle uptake by infiltrating neutrophils can be generalized for different types of NPs is unclear, as the contribution of recruited cells to particle clearance is less well studied.

Our L-IVM imaging revealed that tissue-resident AMs are pivotal in initiating the early inflammatory immune response to inhaled NPs. AM exposure to deposited NPs increased their crawling speed and accumulation at sites of pronounced particle deposition. Previous studies have shown that epithelial deposition of inhaled NPs is not homogeneous, but NPs rather deposit at hotspots within the acinus, the alveolar sacculi, distal to the bronchial-alveolar duct in mice (*98*). Whether AMs get attracted to deposition sites or accumulate there upon random migration remains still unclear. However, we show that AM-mediated NP clearance by phagocytosis is a key event required for neutrophil recruitment. Accordingly, neutrophil extravasation is localized to NP deposition hotspots and AM presence as well. Neutrophil chemoattractants such as CXCL1, CXCL2, and CXCL5 and TNF-α are rapidly released into the airspace upon particle exposure (*7*); however, whether these cytokines initiate leukocyte recruitment or merely amplify the ongoing response has, so far, not been shown. In our study, we identified cellular degranulation and TNF-α signaling as key factors, required for early airspace neutrophilia, implying that TNF-α might be released from NP-phagocytosing AMs. Particle phagocytosis, involving NP recognition via FcγRI and C5aR1, was essential to initiate early lung inflammation, further underscoring the role of AMs as key cells eliciting the response to inert NPs, such as CNPs. While our data implicate AM modulation as the key driver, off-target effects of cromolyn, anti–LFA-1/ICAM-1, anti-C5aR1, and anti-FcγR1 cannot be excluded. Future studies using cell type–specific genetic deletions will be essential to definitively delineate each cell type’s contribution.

In summary, our study demonstrated that alveolar-deposited NPs prompt a rapid inflammatory response that is initiated by intensified AM patrolling behavior and particle phagocytosis. Notably, it was found that the early neutrophil responses are locally focused to those alveoli, which have been challenged by a high burden of deposited NPs. However, only NPs recognized and phagocytosed by AMs induced the immune response, whereas stealth NPs, such as PEGylated QDs, did not. We identified NP uptake by AMs as the key event for the initiation of the pulmonary inflammatory response and shed light on mediators and receptors involved in the initiation of NP-induced sterile inflammation. These insights allow us to propose previously unidentified strategies to regulate AM activity, thus opening potential therapeutic approaches ranging from air pollution and NP exposure to chronic inflammatory lung disease.

## MATERIALS AND METHODS

### Mice

C57BL/6 (WT) mice were purchased from Charles River (Sulzfeld, Germany) for the respective experiments, and Macgreen (Csf1r-EGFP) mice were originally purchased from the Jackson Laboratory (Bar Harbor, ME, USA) and bred in-house. All mice were housed in individually ventilated cages supplied with filtered air in a 12-hour light/12-hour dark cycle within a specific double-barrier, pathogen-free unit at Helmholtz Zentrum München. Mice were fed with autoclaved rodent feed and water ad libitum. All experiments were performed with female animals at 10 to 16 weeks of age. All protocols used were according to the guidelines drafted by the Regierung von Oberbayern (District Government of Upper Bavaria) under the approval number ROB-55.2Vet-2532.Vet_02-19-150.

### Nanoparticles

QDs: Qdot 655 ITK Carboxy (cQD-NP), Qdot 655 ITK Amino (PEG) (aPEG-QDs), and Qtracker (PEG) 655 QDs (PEG-QDs) were purchased from Invitrogen Corporation (Karlsruhe, Germany) as 8 μM solution, or 2 μM in case of PEG-QDs with an emission wavelength of 655 nm. These QDs are composed of a CdSe core encapsulated with a ZnS shell and an extra layer of PEG (PEG-QDs) and with additional amine residues (aPEG-QDs) or without any coating (cQD-NPs). The PEG coating comprises short oligomers (1.3 kDa) (*48*). The core-shell dimensions of the elongated 655-QDs were 10 to 12 nm. The physical characterization of QDs, including biomolecule surface association, was previously performed in our laboratory and has been published (*28*, *48*, *49*).

For biopersistent particles, [Brunauer-Emmett-Teller (BET) or geometric] surface area per mass of lung has been established as an allometrically scalable, biologically relevant dose metric for pulmonary inflammation murine lung weight (0.18 g) (*5*, *99*). For conversion of the typically reported molarity of QD suspensions to this unit, one needs to consider the molar mass (1.5 × 10^6^ to 2 × 10^6^ g/mol), primary geometric particle diameter (20 nm), yielding a mass-specific surface area of (16 cm^2^/g of lung). Printex 90 carbon black (soot with low organic content) NPs (CNP) were purchased from Degussa (Frankfurt, Germany) [diameter, 14 nm; organic content, 1%; mass-specific (BET) surface area, 272 m^2^/g] as described (*100*) and prepared in a stock suspension (5 μg/μl) (in water).

### In vivo labeling of AMs and neutrophils

The method was performed according to a previously published procedure (*26*). PKH26 phagocytic cell labeling kit (Sigma-Aldrich, Burlington, MA, USA) was purchased from Merck KGaA (Darmstadt, Germany). PKH dye stock (0.1 ml; 10^−3^ M) was mixed thoroughly with 0.9 ml of alcohol to make a 100 μM working solution. The working solution was then diluted in Diluent B to prepare 0.5 μM PKH26PCL, serving as the labeling solution for all L-IVM experiments. By oropharyngeal application, mice were given 75 μl of working solution directly into the airway at least 5 days before the experiment.

For in vivo labeling of neutrophils, anesthetized mice [medetomidine (0.5 mg/kg of BW), midazolam (5 mg/kg of BW), and fentanyl (0.05 mg/kg of BW) (MMF), intraperitoneally] were administered with 3 μg of fluorescent-labeled anti-mouse Ly6G antibody (Alexa Fluor 488, clone: 1A8, BioLegend, CA, USA) fluorescent-labeled antibodies via intravenous injection at least 30 min before NP inhalation.

### In vivo blocking/inhibiting experiments

Mice were administered blocking antibodies/isotype control antibodies or inhibitors (see Table 1) via the oropharyngeal method, as described above, in the airways. Three hours after administering blocking antibodies, mice were imaged with L-IVM.

**Table 1.** List of blocking antibodies or inhibitors applied into the airways.

Blocking antibody/inhibitor	Clone	Manufacturer	Dose	RRID
In vivo mAb anti-mouse LFA-1α	M17/4	Bio X Cell, Lebanon, USA	30 μg per mice	AB_1107578
Purified anti-mouse CD54 (ICAM-1) antibody	YN1/1.7.4	Thermo Fisher Scientific, MA, USA	30 μg per mice	AB_467301
Ultra-LEAF purified anti-mouse CD88 (C5aR) mAb	20/70	BioLegend, Fell, Germany	30 μg per mice	AB_2819876
Ultra-LEAF purified anti-mouse CD64 (FcγRI) mAb	W18349F	BioLegend, Fell, Germany	30 μg per mice	AB_2892504
In vivo mAb rat IgG2a isotype control	2A3	Bio X Cell, Lebanon, USA	30 μg per mice	AB_1107769
Ultra-LEAF purified rat IgG2b, κ isotype Ctrl antibody	RTK4530	BioLegend, Fell, Germany	30 μg per mice	AB_11147763
Purified rat IgG1, κ isotype control antibody	RTK2071	BioLegend, Fell, Germany	30 μg per mice	AB_326508
Purified anti-mouse TNF-α antibody	MP6-XT22	BioLegend, Fell, Germany	30 μg per mice	AB_315423
Cromolyn sodium salt		Sigma-Aldrich, Burlington, MA, USA	0.2 mg/kg (BW)	

Mice were pretreated with blocking antibodies/isotype control antibodies or stabilizers (Table 2) applied into the blood circulation via intravenous injection. Thirty minutes after administering blocking antibodies, mice were exposed to NPs.

**Table 2.** List of blocking antibodies or stabilizers applied into the blood circulation.

Blocking antibody/inhibitor	Clone	Manufacturer	Dose	RRID
In vivo mAb anti-mouse LFA-1α	M17/4	Bio X Cell, Lebanon, USA	30 μg per mice	AB_1107578
Purified anti-mouse CD54 (ICAM-1) antibody	YN1/1.7.4	Thermo Fisher Scientific, MA, USA	30 μg per mice	AB_467301
Cromolyn sodium salt		Sigma-Aldrich, Burlington, MA, USA	0.2 mg/kg (BW)	

### Lung intravital microscopy

The lung intravital microscope, based on the VisiScope A1 imaging system (Visitron Systems GmbH, Puchheim, Germany), is equipped with a light-emitting diode (LED) light source for fluorescence epi-illumination (CoolLed p-4000, Andover, UK). QDs were excited using the 385-nm LED module, melamine resin particles for blood flow tracing were illuminated with the 655-nm LED module, anti–Ly6G-488 was exited with 470 nm, and PKH26 with 550 nm (all at 50% output power and exposure time of 50 ms). The light was directed onto the sample via a quad-band filter [F66-014, 4′,6-diamidino-2-phenylindole (DAPI)/fluorescein isothiocyanate/Cy3/Cy5 Quad LED ET Set, AHF Analysentechnik AG, Tuebingen, Germany]. Microscope images were acquired by a water dipping objective [20×, numerical aperture (NA) of 1.0; Zeiss MicroImaging GmbH, Jena, Germany). A beam splitter (T 580 lpxxr Chroma Technology Corp., Bellows Falls, USA) was used to divide the light from the sample. Images were acquired with two Rolera EM2 cameras and VisiView Imaging software (Visitron Systems GmbH, Puchheim, Germany). The experimental approach was adapted from previously described work (*27*). Mice were deeply anesthetized with a mixture of MMF by intraperitoneal injection, maintained at 37°C body temperature, and monitored using the small animal physiological monitoring (Harvard Apparatus, MA, USA). Surgical sites (neck and left chest) were locally anesthetized with Bucaine (50 μg per site; Puren Pharma, Germany). Tracheostomy was performed, and a small blunt catheter (20 gauges; B. Braun, Melsungen, Germany) was threaded <5 mm into the trachea and connected to a small rodent ventilator (MiniVent, Harvard Apparatus, MA, USA). Mice were ventilated with a stroke volume (tidal volume) of 10 μl/g of BW and 150 breaths/min under positive end-expiratory pressure (0.1 cm/g of BW) with 100% oxygen.

The mice were then placed in the right lateral decubitus position, and a custom-made flanged thoracic suction window was inserted into a 5-mm intercostal incision through the parietal pleura between ribs 3 and 4 of the left chest. Suction (20 to 25 mmHg) was used to immobilize the lung by a custom-made system consisting of a differential pressure gauge (Magnehelic, Dwyer Instruments Inc., USA) and a negative pressure pump (Nupro, Willoughby, USA). Anesthesia was maintained by the administration of half the first dose of MMF (intraperitoneally) every 45 min.

### NP aerosol inhalation

For dose-controlled aerosol inhalation, the respiratory parameters of the mechanically ventilated mouse were set to 15 μl/g and 150 breaths/min (MiniVent, model 845, Harvard Apparatus, USA). Subsequently, 20 μl of 4 μM (=7 μg/μl) QD suspension (1:2 diluted stock suspension; diluent:distilled water with 1% saline) or CNP (stock; 5 μg/μl) suspension were nebulized with a breath-activated vibrating mesh nebulizer [Aeroneb Lab Small, volume-weighted droplet diameter = 2.5 to 4.0 μm; manufacturer information VMD Aerogen Inc., Ireland]. For this, an infrared-based motion detector was installed in front of the moving ventilator piston for triggering nebulizer activation for 20 ms per breath at the onset of the inhalation phase. After the NP inhalation procedure that typically lasted 1 min for QDs and 5 min for CNPs, the tidal volume was set back to normal breathing conditions (10 μl/g of BW and 150 breath/min) as used for L-IVM.

### Measurement of blood flow velocity

Melamine resin fluorescent particles (microParticles, Berlin, Germany) (50 μl of 0.05% stock solution) were injected (intravenously) into mice and imaged at ~2.5 frames/s using the fast acquisition mode. The pulmonary blood flow velocity of mice was represented by the trajectory velocity of melamine formaldehyde fluorescence particles. The “Manual Tracking” function of ImageJ (National Institutes of Health, Bethesda, USA) was applied to give a specific coordinate axis to the tracing bead location at each time point. Then, bead velocity was analyzed with the Chemotaxis and Migration Tool software (ibidi GmbH, Gräfelfing, Germany)

### Quantification of neutrophil kinetics

To quantify neutrophil numbers in the pulmonary microcirculation and the alveolar region, in vivo images of seven observation areas were randomly recorded at intervals of 15 or 30 min pre- and postinhalation. The L-IVM images were analyzed with ImageJ software (National Institutes of Health, Bethesda, USA) to quantify the neutrophil numbers. For neutrophil counting, the “Trackmate” function of ImageJ was used. All neutrophils were automatically counted by the software using the −15 min (neutrophil baseline) image for the threshold and size setting. The same settings were used for subsequent time points, and the results underwent manual calibration. Neutrophil and AM motility tracked with Trackmate were analyzed, including cell velocity and directness and visualized using the Manual Chemotaxis tool (https://ibidi.com/img/cms/downloads/in/Manual_ChemotaxisTool_2_0_eng.pdf).

### BAL preparation and cell differentiation

Mice were anesthetized via intraperitoneal injection of xylazine (5.5 μg/g of BW; WDT, Garbsen, Germany) and ketamine (0.5 mg/g of BW; PharmaWiki, Disentis, Switzerland) and euthanized by abdominal aorta exsanguination. Immediately after, BAL was performed by trachea intubation with a 20-gauge cannula (Braun, Melsungen, Germany) and by infusing the lungs eight times with 1.0 ml of sterile phosphate-buffered saline (PBS). The total recovered volume was around 8 ml per mouse. Cell pellets obtained after centrifugation (400*g* for 20 min at 4°C) were resuspended in 1 ml of sterile PBS, and the cell amount was counted using the trypan blue exclusion method. BAL cell differentials were accomplished on cytospin slides with May-Grünwald-Giemsa staining (3 × 200 cells counted).

### Ex vivo 3D light sheet fluorescence imaging of the lung

C57BL/6 or Csf1r-EGFP mice were euthanized by exsanguination and transcranial perfused with 30 ml of sterile PBS at room temperature to flush all blood (EGFP-labeled cells included) from the lungs. After that, only EGFP-expressing AMs and interstitial macrophages are left in the lung. The lung tissue was stained and cleared as described earlier (*98*). Briefly, samples were fixed in 4% paraformaldehyde (PFA) at 4°C overnight. Next, samples were incubated in PBSG-T (0.2% gelatin, 0.01% thimerosal, and 0.5% Triton X-100 in PBS) for 3 days with rotation (70 rpm) at room temperature to block nonspecific antibody binding. Lung samples then were incubated with an anti-mouse GFP antibody (clone: ab13970, Abcam, Waltham, USA), which is diluted in 0.1% Saponin in PBSG-T for 7 days with rotation (70 rpm) at 37°C. Afterward, lung samples were washed six times with PBST (0.5% Triton X-100 in PBS) for 1 hour each and incubated with goat anti-chicken (Alexa Fluor 647) secondary antibody (ab150175, Abcam, Waltham, USA) and DAPI diluted in 0.1% Saponin in PBSG-T for 3 days with rotation (70 rpm) at 37°C. Samples were washed six times again in PBST for 1 hour each with rotation at room temperature to finish the last step of staining. Subsequently, clearing was performed after dehydration in a concentration gradient of tetrahydrofuran [THF; Sigma-Aldrich, Burlington, MA, USA; 50% (v/v) THF/H_2_O overnight, 50% THF/H_2_O for 1 hour, 80% THF/H_2_O for 1 hour, 100% THF for 1 hour, 100% THF overnight, and 100% THF for 1 hour] with continuous gentle shaking. Next, samples were incubated in dichloromethane (Sigma-Aldrich, Burlington, MA, USA) for around 30 to 40 min and eventually immersed in dibenzyl ether (DBE; Sigma-Aldrich, Burlington, MA, USA) at least 2 hours before imaging. Imaging was performed in DBE with an light sheet fluorescence microscope (UltraMicroscope II, LaVision Biotec) equipped with an scientific complementary metal-oxide semiconductor camera (Andor Neo, Abingdon, UK) and a 2× objective lens (Olympus MVPLAPO 2×/0.5 NA) equipped with an Olympus MVX-10 zoom body, which provided zoom-out and zoom-in ranging from 0.63× to 6.3×. Light sheet images were generated with different magnification factors and a step size of 5 to 10 μm according to sample size with 470 ± 30/640 ± 30–nm excitation/emission (ex/em) bandpass filters for QDs. Lung tissue autofluorescence was generally scanned with 520 ± 40/585 ± 40–nm ex/em filters to show the microstructure of the lungs. One hundred–millisecond exposure time and 95% laser power are typically set with LSFM, where the light sheet has a different *xy* width and NA to match the different sample sizes. During the LSFM image acquisition, the samples were immersed in DBE. Imaris 9.1.0 (Bitplane, Belfast, UK) was used to perform 2D and 3D rendering and image processing.

### Fluorescence-based analysis of QD-NP dose in lung homogenates

Mice were euthanized immediately after L-IVM experiments by exsanguination, and then the lung perfusion was performed following the above protocol. Subsequently, the whole lungs were removed. Quantification of QDs was based on a previously published protocol (*101*). Briefly, each lung was immersed in 1 ml of tissue lysing solution (SOLVABLE, PerkinElmer, Waltham, USA) at 50°C for 24 hours until complete tissue dissolution. Subsequently, the QD fluorescence intensity of the samples was measured with a spectrofluorometer (ex/em wavelength, 400/655 nm; Safire 2, Tecan, Zürich, Switzerland) and compared to standard curves, which were generated for each QD type by measuring solubilized blank lung samples with known but different amounts of QDs.

### Multiplex cytokine/chemokine analysis

Cytokines and chemokines in BAL fluid were measured using the multiplex bead array system Bio-Plex Pro Mouse Chemokine Assay Panel 31-Plex (#12009159, Bio-Rad Laboratories GmbH), following the manufacturer’s instructions. Data were acquired using a Luminex 200 system with Bio-Plex Manager 6.1 software. Standard curves were fitted using the logistic-5PL regression type. The data were visualized with a heatmap generated using the statistical programming environment R (v4.4.4) using the heatmap package (v1.0.12).

### Ex vivo whole lung imaging

To obtain a spatial distribution of fluorescent nanomaterials throughout the lungs, epifluorescence imaging with an in vivo imaging system (IVIS) (Lumina II, Caliper/PerkinElmer, USA) was used for (nontissue cleared) lungs. Briefly, the isolated lungs were placed on a platform located in the center of the IVIS chamber and imaged with QD-specific ex/em filters (ex/em, 475 nm/Cy5.5; exposure time, auto; binning, medium; F/stop: 16; lamp level, high). The fluorescence/white-light images were acquired with the Living Imaging 4.0 software (Caliper, Newton, MA, USA) to determine fluorescence intensity. Because of the lack of tissue clearing, the obtained fluorescence profile is only an estimate of the QD distribution in the detector-facing, peripheral layer of the lung. However, for the spatially uniform aerosol inhalation, this is a good estimate of QD distribution in the lung.

### Transmission electron microscopy

TEM images of lung tissue were acquired as previously described (*102*). Briefly, lungs were perfusion-fixed with 2% formaldehyde and 2% glutaraldehyde in 0.1 M cacodylate buffer (pH 7.4) via the left ventricle. Last, the lung was also filled with fixative via the trachea. After dissection, the samples were fixed for 3 hours at room temperature. The tissue was cut into smaller cubes (1 mm^3^) and postfixed in 1% OsO_4_ containing 1.5% potassium cyanoferrate. After dehydration, the samples were embedded in epon. Ultrathin (70 nm) sections were cut in serial sections (UC6 ultramicrotome, Leica, Vienna, Austria). Except for some sections that were counterstained with uranyl acetate and lead, sections were not stained so that QDs were unambiguously identified by their morphology. The images were acquired at 80 kV on an FEI Tecnai 12 transmission electron microscope (Thermo Fisher Scientific). Representative areas were imaged with a charge-coupled device camera (Veleta, EMSIS, Muenster, Germany).

### MH-S cell culture

Murine MH-S AM–like cells were purchased from the American Type Culture Collection (RRID: CVCL_3855, CRL-2019, ATCC, Manassas, USA) and grown in RPMI 1640 medium with supplements containing 0.05 mM 2-mercaptoethanol, 10% fetal bovine serum (FBS), and 1% penicillin-streptomycin at 37°C and 5% CO_2_.

### QD delivery to MH-S cells

A total of 500,000 MH-S cells were distributed in 100 μl of cell culture medium and then seeded on the apical side of a six-well transwell insert (4.2 cm^2^, 0.4-μm pores; Corning, NY, USA) placed in a well with 1.7 ml of basal medium. At 24 hours after cell seeding, the basal medium was changed with the aspiration of the 100-μl apical medium, and then the cells were equilibrated at ALI conditions for 2 hours. Then, the cells were exposed to the QD aerosol droplets under the ALI conditions using the VITROCELL Cloud Alpha 6 aerosol-cell exposure system. For this, 100 μl of 1:20 diluted QD suspension in distilled water with 0.1% NaCl (0.7 μg of QD/μl) was nebulized with an Aeroneb Pro nebulizer (Aerogen Inc., Ireland), and ~5 μl per insert is deposited directly onto the cells (*50*). Thereafter, cells were incubated for 2 hours and washed with PBS to remove the unattached particles after incubation. IF staining imaging was used for the qualitative comparison of NP phagocytosis by MH-S across different groups, while FACS was used for the quantitative analysis of NP phagocytosis by MH-S in these groups. For IF staining, MH-S cells were fixed with 4% PFA for 10 min, followed by staining with DAPI (1 μg/ml; Roche, Basel, Switzerland) and Alexa Fluor 488-labeled phalloidin (5 U/liter; A12379, Thermo Fisher Scientific, Carlsbad, USA) for 30 min. The cells were then washed three times with Dulbecco’s PBS (DPBS) containing 0.1% Tween 20, each wash lasting 5 min. Last, the stained samples were mounted using Dako fluorescence mounting medium (Dako Omnis, Agilent, Santa Clara, USA) and covered with coverslips. For FACS, after washing in the FACS buffer (2% bovine serum albumin and 1 mM EDTA in PBS), cells were collected and analyzed using a BD FACSCanto II flow cytometer (BD Biosciences, NJ, USA) with BD FACSDiva software.

### WST cell viability assay

MH-S cells were seeded in 24-well plates at a density of 10^5^ per well and cultured overnight. Cells were exposed to QD particles (8 nM) and vehicle as a control in 500 μl of cell culture medium in submerged conditions for 1 hour. The supernatant was removed, and the cells were incubated with 10% WST reagent (Roche, Basel, Switzerland) for 15 min at 37°C and 5% CO_2_. Next, WST samples were collected and centrifuged at 14,000 rpm for 10 min at room temperature. Absorbance at 450 nm of 200 μl solution was measured in 96-well plates with a plate reader (Tecan Trading AG, Männedorf, Switzerland). Each sample was measured from two independent wells. The absorbance value was corrected with a blank sample (WST solution nonincubated with cells). The cell viability of each sample was compared with the control group according to the following equation: cell viability = [sample optical density (OD) − blank OD] / (control OD − blank OD) × 100%.

### Phagocytosis assay

MH-S AM–like cells were seeded in 24-well plates at a density of 25 × 10^4^ cells per well. Cells were pretreated with anti–ICAM-1, anti–LFA-1, anti-CD44, anti-CD64, anti-CD88, and isotype (10 μg/ml) antibodies, cromolyn (4 μg/ml; C0399, Merck, Germany) or vehicle control, respectively, at 37°C for 3 hours at 5% CO_2_ (Table 3). Thereafter, pHrodo Green *E. coli* BioParticles (5 μg/ml; Thermo Fisher Scientific, Carlsbad, USA), pH-dependent fluorescent particles, were added to the supernatant evenly for 1 hour. After washing in the FACS buffer, cells were collected and analyzed using a BD FACSCanto II flow cytometer (BD Biosciences, NJ, USA) with BD FACSDiva software.

**Table 3.** List of blocking antibodies or inhibitors applied in vitro.

Blocking antibody/inhibitor	Clone	Manufacturer	Dose	RRID
In vivo mAb anti-mouse LFA-1α	M17/4	Bio X Cell, Lebanon, USA	10 μg/ml	AB_1107578
Purified anti-mouse CD54 (ICAM-1) antibody	YN1/1.7.4	Thermo Fisher Scientific, MA, USA	10 μg/ml	AB_467301
Ultra-LEAF purified anti-mouse CD88 (C5aR) mAb	20/70	BioLegend, Fell, Germany	10 μg/ml	AB_2819876
Ultra-LEAF purified anti-mouse CD64 (FcγRI) mAb	W18349F	BioLegend, Fell, Germany	10 μg/ml	AB_2892504
In vivo mAb rat IgG2a isotype control	2A3	Bio X Cell, Lebanon, USA	10 μg/ml	AB_1107769
Ultra-LEAF purified rat IgG2b, κ isotype Ctrl antibody	RTK4530	BioLegend, Fell, Germany	10 μg/ml	AB_11147763
Cromolyn sodium salt		Sigma-Aldrich, Burlington, MA, USA	4 μg/ml	

### In vitro cQD-NP uptake

MH-S cells were seeded at 10^5^ cells per well in 24-well plates for 2 days and then treated with anti–ICAM-1, anti-CD88, anti-CD64, anti–immunoglobulin G2a (IgG2a) isotype, or anti-IgG2b isotype antibodies (10 μg/ml each), or vehicle, at 37°C for 3 hours at 5% CO_2_. Samples were incubated with 4 nM cQD-NPs in medium for 90 min. For IF staining, cells were fixed in 4% PFA for 10 min, stained with DAPI (1 μg/ml; Roche, Basel, Switzerland) for 30 min, washed 3× with DPBS (5 min each), and mounted with Dako medium (Dako Omnis, Agilent, Santa Clara, CA, USA). Imaging used Alexa Fluor 568 (cQD-NPs) and DAPI (nuclei) channels. Cell segmentation used Imaris 10.1.0 Cells function (Oxford Instruments, Abingdon, UK), using DAPI and cQD-NP channels to calculate mean cQD-NP intracellular fluorescence intensities.

### Inhibition of cytokine release by THP-1 cells using cromolyn

Three milligrams of Printex 90 carbon black NPs were weighed and subjected to thermal treatment at 200°C for 2 hours to ensure sterility. The particles were then suspended in complete RPMI 1640 culture medium (Thermo Fisher Scientific, USA) supplemented with 10% FBS (Thermo Fisher Scientific, USA), 1% antibiotic-antimycotic solution (Thermo Fisher Scientific, USA), and 0.05 mM β-mercaptoethanol (Thermo Fisher Scientific, USA) to achieve a stock concentration of 1 mg/ml. To ensure adequate dispersion, the suspension underwent sonication in an ice-cold water bath for 5 min, followed by sonication using a VCX 750 sonicator (Sonics & Materials, CT, USA) at 750 W, 30% amplitude, and continuous pulse for 30 s. Immediately before cellular exposure, particle suspensions were briefly vortexed. Monodisperse cQD-NPs were diluted to a working concentration of 1:100 in complete culture medium by vigorous vortexing. A 100 mM stock solution of cromolyn sodium salt (C0399, Merck, Germany) was prepared by dissolving 205 mg in 4 ml of PBS. Working dilutions were subsequently prepared in complete culture medium.

The human monocytic leukemia cell line THP-1 (RRID: CVCL_0006, ATCC-TIB-202, Manassas, USA) was cultured in RPMI 1640 medium supplemented as described above and maintained at 37°C in a humidified atmosphere with 5% CO_2_. For differentiation into macrophages, THP-1 cells were seeded in 96-well plates and treated with 100 nM phorbol 12-myristate 13-acetate (Sigma-Aldrich, USA) for 48 hours. Following differentiation, the supernatant was carefully removed, and the adherent macrophages were washed once with tempered PBS to eliminate any remaining undifferentiated monocytes. Fresh complete medium containing FBS was then added. THP-1 macrophages were preincubated with cromolyn sodium at concentrations of 0.5, 5, and 10 μM for 30 min. Subsequently, cells were exposed to cQD-NPs at final dilutions of 1:1000 and 1:2000 or to CNPs at concentrations of 50 or 100 μg/ml for either 90 min or 24 hours at 37°C. Cellular activity was assessed using the WST-1 assay. Following particle exposure, wells were washed once with tempered PBS. A 10% WST-1 solution (catalog no. 11644807001, Sigma-Aldrich, USA) in complete medium (200 μl) was added to each well, and the plates were incubated for 1 hour at 37°C with 5% CO_2_. To avoid potential interference from CNPs, 100 μl of the supernatant from each well was carefully transferred to a new 96-well plate, and the absorbance was measured using a SpectraMax iD3 microplate reader (Molecular Devices, USA). The release of TNF-α into the cell culture supernatants was quantified using an enzyme-linked immunosorbent assay (ELISA) kit (Human TNF-α DuoSet ELISA, catalog no. DY210, R&D Systems) according to the manufacturer’s instructions.

### Ana-1 macrophage culture

The mouse Ana-1 macrophage cell line (RRID: CVCL_0142, 305172, CLS, Nanterre, France) was used in the study (*103*). Ana-1 cells were cultured in RPMI 1640 medium (Gibco, Grand Island, NY, USA) supplemented with 15% FBS (PAN Biotech, Aidenbach, Germany), 2 mM l-glutamine (Gibco, Grand Island, NY, USA), 1% nonessential amino acids (Gibco, Grand Island, NY, USA), and penicillin (100 U/ml) and streptomycin (100 μg/ml) (Gibco, Grand Island, NY, USA). For in vitro experiments, Ana-1 cells were exposed to CNP (50 μg/ml), LPS (1 μg/ml), or left untreated as a control group. Samples were harvested after 3 and 9 hours.

### RNA isolation and transcriptomics

Whole-cell RNA from Ana-1 cells after 3- and 9-hour exposure was isolated with the NucleoSpin RNA Plus kit (MACHEREY-NAGEL, Duren, Germany) following the instructions of the manufacturer. Subsequently, the Agilent 2100 Bioanalyzer was applied to assess RNA quality for following quantification with Mouse Clariom S arrays (Thermo Fisher Scientific, Waltham, MA, USA). The detailed description of this approach was published previously (*75*). Statistical analysis was performed using the statistical programming environment R (v4.0.4). Differentially expressed genes analysis were performed by limma *t* test (*P* < 0.05). To visualize the expression level of a cluster of genes, heatmaps were generated in R with the pheatmap package. Pathway analysis was performed by clusterProfiler package, comparing the Hallmark database, and bar plot was used to visualize enriched terms (ggplot2 package). Array data used in the study have been submitted to the Gene Expression Omnibus (GEO) database at National Center for Biotechnology Information (NCBI) via the accession number: GSE223818.

### Single-cell transcriptomics

Mouse lung single-cell data were downloaded under GEO accession: GSE185006, six lungs from mice exposed to filtered air were included in the study to represent healthy lungs. Mouse lung cell type annotation and visualization were performed as described (*104*). Analysis was performed using Scanpy (v1.8.0). The visualization of dimension-reduced single-cell transcriptomic data was performed by Uniform Manifold Approximation and Projection.

### Statistical analysis

All data were presented as means ± SEM and plotted with GraphPad Prism 10 (GraphPad Software Inc., La Jolla, USA), with the sample sizes and the number of repeats indicated in the figure legends. Comparison of results between two groups for normally distributed data was analyzed using the two-sided Student’s *t* test and for nonparametric data with the Mann-Whitney rank-sum test. Comparisons among multiple groups were performed using a one-way analysis of variance (ANOVA) with Tukey’s comparisons test. Significances are defined as *P < 0.05, **P < 0.01, ***P < 0.001, and ****P < 0.001, while *P* ≥ 0.05 was considered not significant (ns).
