# Supplementary material for: Alveolar macrophages initiate the spatially targeted recruitment of neutrophils after nanoparticle inhalation
Source: Sci Adv. 2025 Nov 7;11(45):eadx8586. doi: 10.1126/sciadv.adx8586 (PMC12594175; doi:10.1126/sciadv.adx8586)
Supplement: Supplementary file 1 — Figs. S1 to S8 [file sciadv.adx8586_sm.pdf]

Science Advances
AAAS

# Supplementary Materials for

## Alveolar macrophages initiate the spatially targeted recruitment of neutrophils after nanoparticle inhalation

Qiongliang Liu *et al.*

Corresponding author: Qiongliang Liu, qiongliang.liu@shgh.cn; Markus Rehberg, markus.rehberg@helmholtz-munich.de

*Sci. Adv.* **11**, eadx8586 (2025)
DOI: 10.1126/sciadv.adx8586

**This PDF file includes:**

Figs. S1 to S8

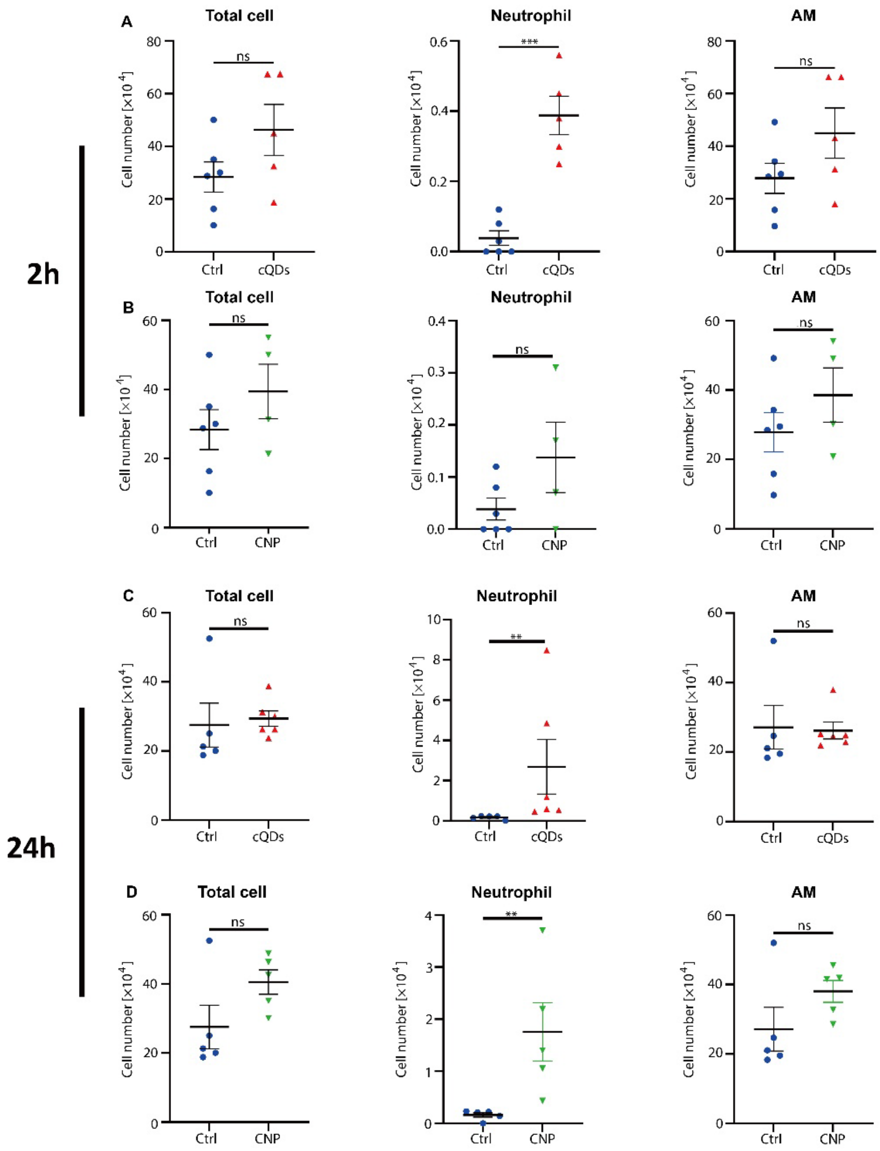


**Fig. S1. Bronchiolar-alveolar-lavage cell numbers at 2 hours and 24 hours after cQDs/CNP inhalation.**
(**A**) and (**B**) 2 h and (**C**) and (**D**) 24 h after NP inhalation the numbers of total cells, AMs and neutrophils were determined in May Grunwald-stained-cytospin samples of cQD and CNP exposed mice (16 cm2/g cQDs; 170 cm2/g CNP), respectively, and compared to vehicle controls.
Data are presented as means ± SEM, $n$= 4-6 mice/group, ns: $p \geq 0.05$, *: $p < 0.05$, **, $p < 0.01$ and ***, $p < 0.001$ by paired Student's t-test (parametric data) or Mann-Whitney rank-sum test (nonparametric data).

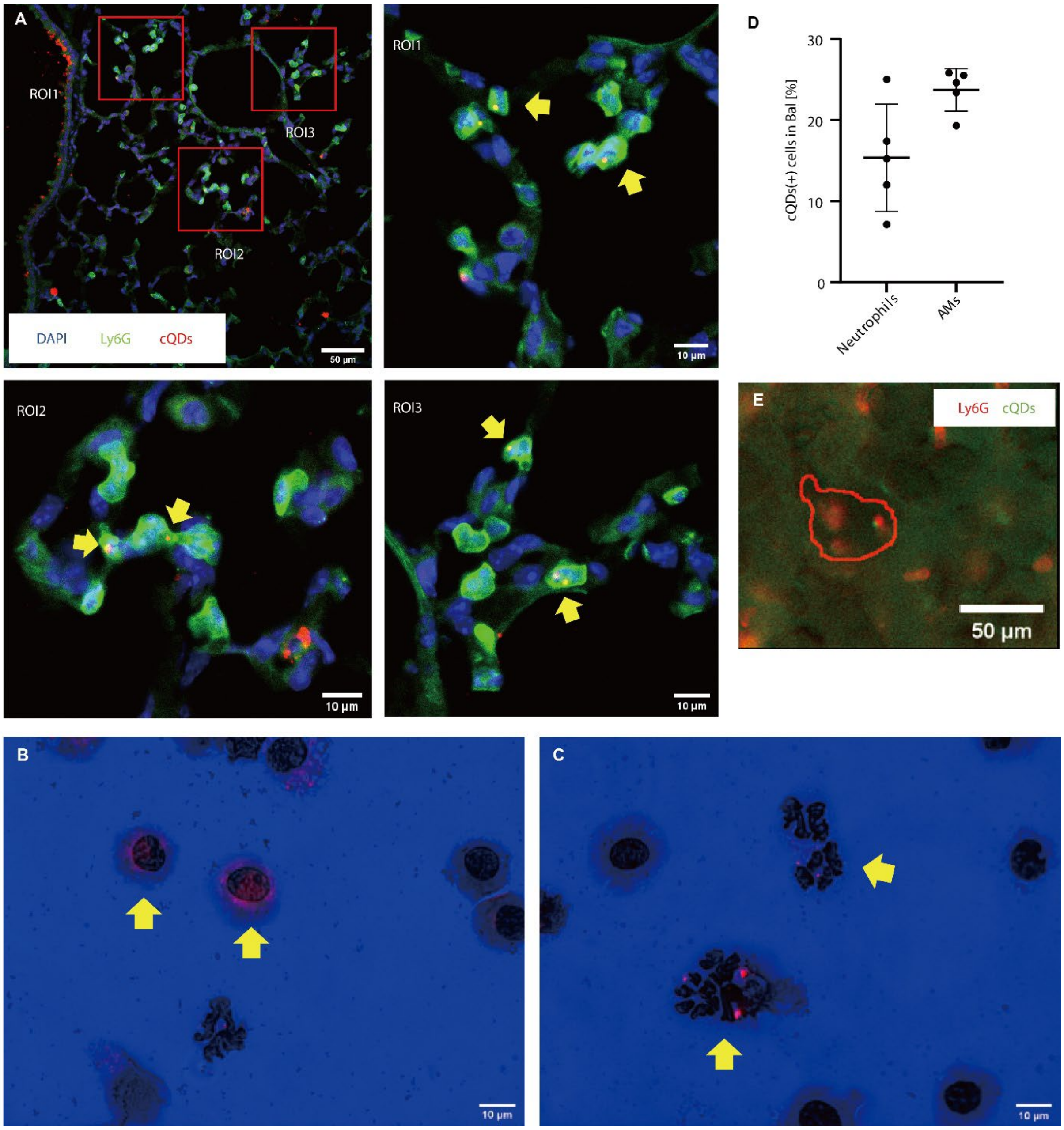


**Fig. S2. Infiltrating neutrophils internalize nanoparticles in the alveoli.**

**A**) Fluorescence confocal microscopy of WT mice lung slices obtained 90 min after cQDs (red) inhalation. Neutrophils labeled with anti-Ly6G mAb (green). Nuclei stained by DAPI (blue) (Scale bar: 50 µm). Frequently alveolar localized neutrophils are colocalized with cQD fluorescent spots, indicating cQDs cellular uptake. ROI1-ROI3: Magnifications highlight cQDs-laden neutrophils (arrows), scale bars: 10 µm.

(**B**) and (**C**) cQDs (red) can be detected in AMs (b, arrows) as well as in neutrophils (**C**, arrows) in May Grunwald-stained BAL cytospin-samples, collected at 24 h after cQD exposure, by combined epifluorescence and phase contrast imaging (Scale bars: 10 µm).

(**D**) Quantification of cQD positive AMs and neutrophils in BAL samples obtained 24 h after cQDs inhalation, *n*=4 mice.

(**E**) L-IVM image at 60 min after cQDs-inhalation shows cQD colocalization with alveolar localized neutrophils (cQDs: green, neutrophils labeled by anti-Ly6G mAbs (*i.v.*): red, scale bar: 50 µm).

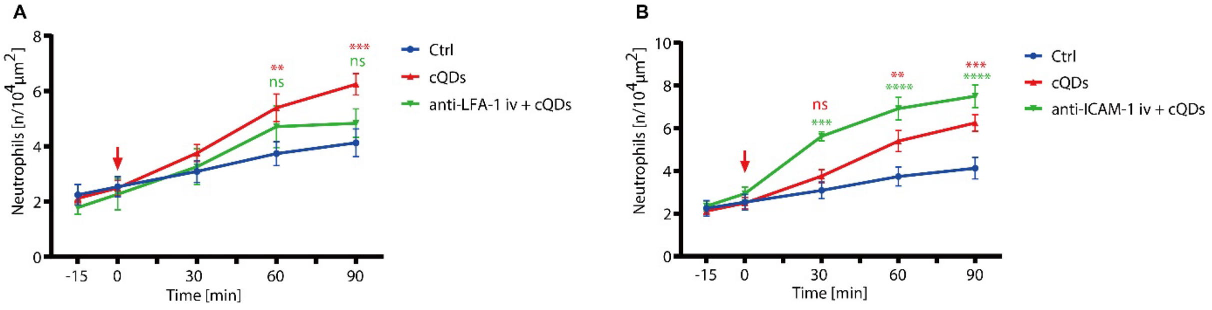


**Fig. S3. Blockade of intravascular LFA-1 as well as ICAM-1 has no mitigating effect on cQDs-evoked recruitment of neutrophils.**

(**A, B**) Alterations in neutrophil numbers in the alveolar region over 90 min were analyzed by L-IVM in mice receiving LFA-1 or ICAM-1 blocking mAbs via intravenous injection 30 min prior to inhalation of cQDs or vehicle-control, *n*=3 mice/group.

Data are presented as Means ± SEM. ns: $p \geq 0.05$, *: $p < 0.05$, **: $p < 0.01$, ***: $p < 0.001$ and ****: $p < 0.0001$.

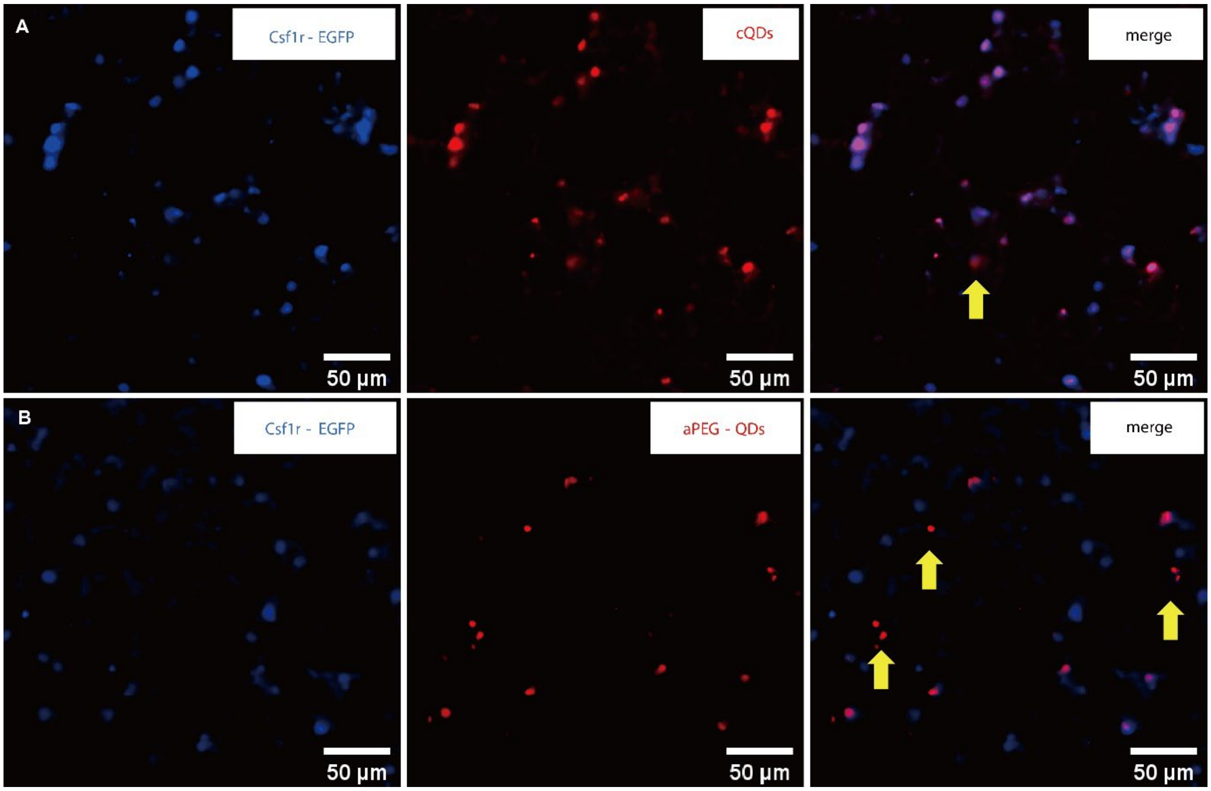


**Fig. S4. PEGylation prevents QD NPs from being internalized by AMs.**

(**A, B**) Tissue clearing was used to achieve co-imaging of AMs from Macgreen mice (Csf1r-EGFP is expressed selectively in macrophage and monocyte cell lineages) after transcranial perfusion for flushing out all Csf1r-EGFP labeled cells in the blood. 2D images from lightsheet fluorescence microscope images of cQDs and aPEG-QDs exposed mice (2 h). Csf1r-1(+) AMs are shown in blue, QDs are shown in red, AMs with ingested QDs ingested appear in purple. (panel A: cQDs, panel B: aPEG-QDs, Scale bar: 50 µm).

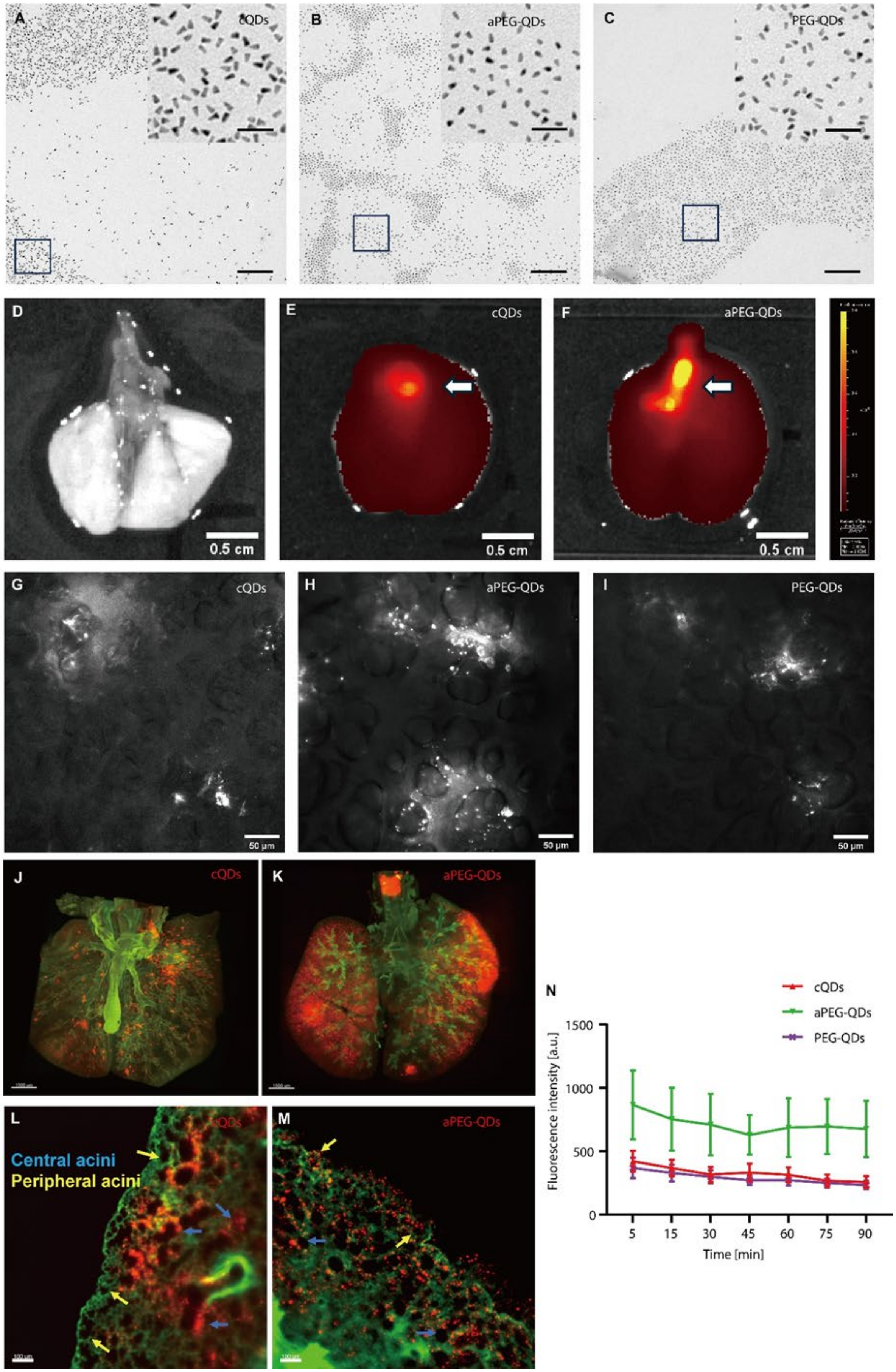

A
cQDs
B
aPEG-QDs
C
PEG-QDs
D
0.5 cm
E
cQDs
0.5 cm
F
aPEG-QDs
0.5 cm
G
cQDs
50 μm
H
aPEG-QDs
50 μm
I
PEG-QDs
50 μm
J
cQDs
K
aPEG-QDs
L
cQDs
Central acini
Peripheral acini
M
aPEG-QDs
N
cQDs
aPEG-QDs
PEG-QDs
Fluorescence intensity [a.u.]
1500
1000
500
0
5
15
30
45
60
75
90
Time [min]

**Fig. S5. 3D/2D mapping of QD distribution in entire mouse lungs upon ventilator-assisted QD-NP aerosol inhalation.**

(**A**-**C**) Electron microscopic images of the three different QD-species. QDs were diluted in distilled water (panel A: cQDs, panel B: aPEG-QDs and panel C: PEG-QDs, scale bars: 200 nm).

(**D**-**F**) Analysis of cQD and aPEG-QD distribution in whole lungs using epifluorescence imaging. Typical ex vivo lung images from mice at 2 h after receiving 16 cm2/g (geom-surface area of NPs / mass-lung) cQDs or aPEG-QDs via inhalation and vehicle control using the IVIS system, which is particularly sensitive to the most peripheral part of the lung which is the focus of L-IVM analysis (panel D: Ctrl, panel E: cQDs, panel F: aPEG-QDs).

(**G-I**) L-IVM images of the three types of QDs (G: cQDs; H: aPEG-QDs; I: PEG-QDs) showing the QD distribution pattern in the most peripheral alveolar part of the lungs 5 min after pulmonary NP inhalation. QDs are shown in white. Scale bars: 50 µm.

(**J-M**) The distribution of QDs (red) in the Z-stack images of entire cleared lungs obtained by light sheet microscopy (panel J and K: 3D MIP, L: cQDs and M: aPEG-QDs, scale bar: 1500 µm) and in single z-planes (panel L and M: 2D xy slice, L: cQDs and M: aPEG-QDs, scale bar: 200 µm) of the tissue structure (autofluorescence, green) 1h after inhalation of different types of (16 cm2/g) QDs.

(**N**) Average QD fluorescence intensities in L-IVM images at consecutive time points after inhalation of cQDs, aPEG-QDs, or PEG-QDs (Means ± SEM, *n*= 3 mice/group)

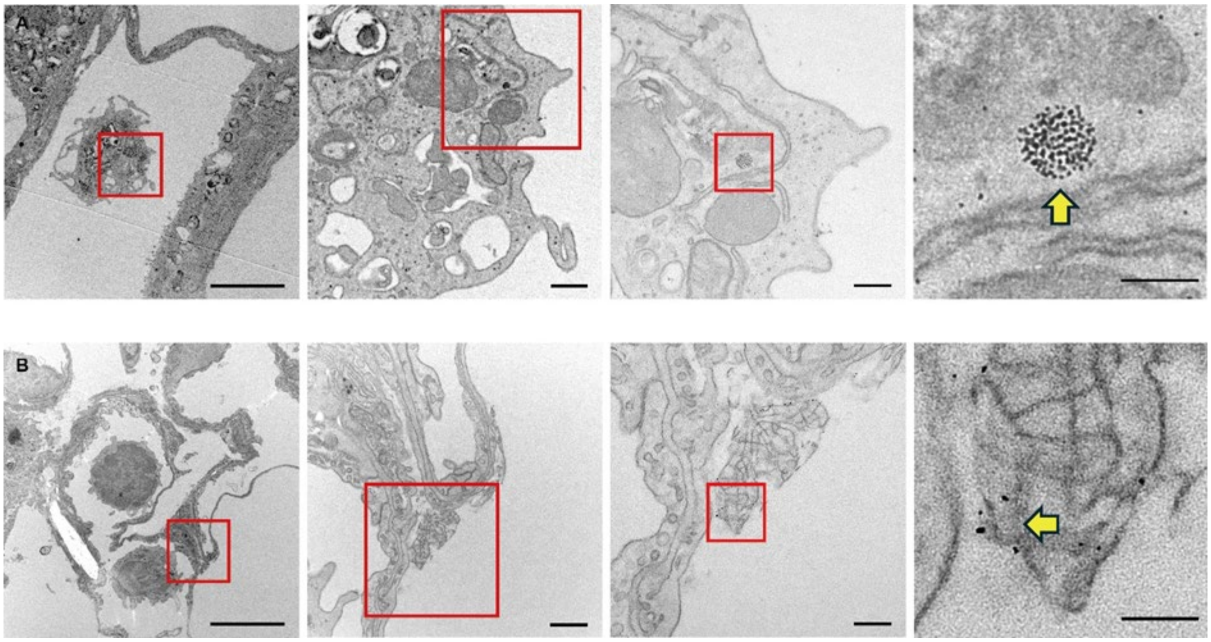


**Fig. S6. cQDs accumulate in AMs and are attached to surfactant lattices.**

Transmission electron microscopy of cQDs-exposed lungs (1 h).

(**A**) Aggregates of cQDs were found within the cytoplasm and endolysosomes of AMs.

(**B**) Non-cell associated "free" cQDs can also be found attached to surfactant components (tubular myelin sheaths) at the alveolar air-liquid interface. QDs are visible as distinct black points (yellow arrows) within the surfactant lattice. Overview and detailed higher magnification pictures of depicted areas with corresponding scale bars: 5 µm, 500 nm, 200 nm, 100 nm.

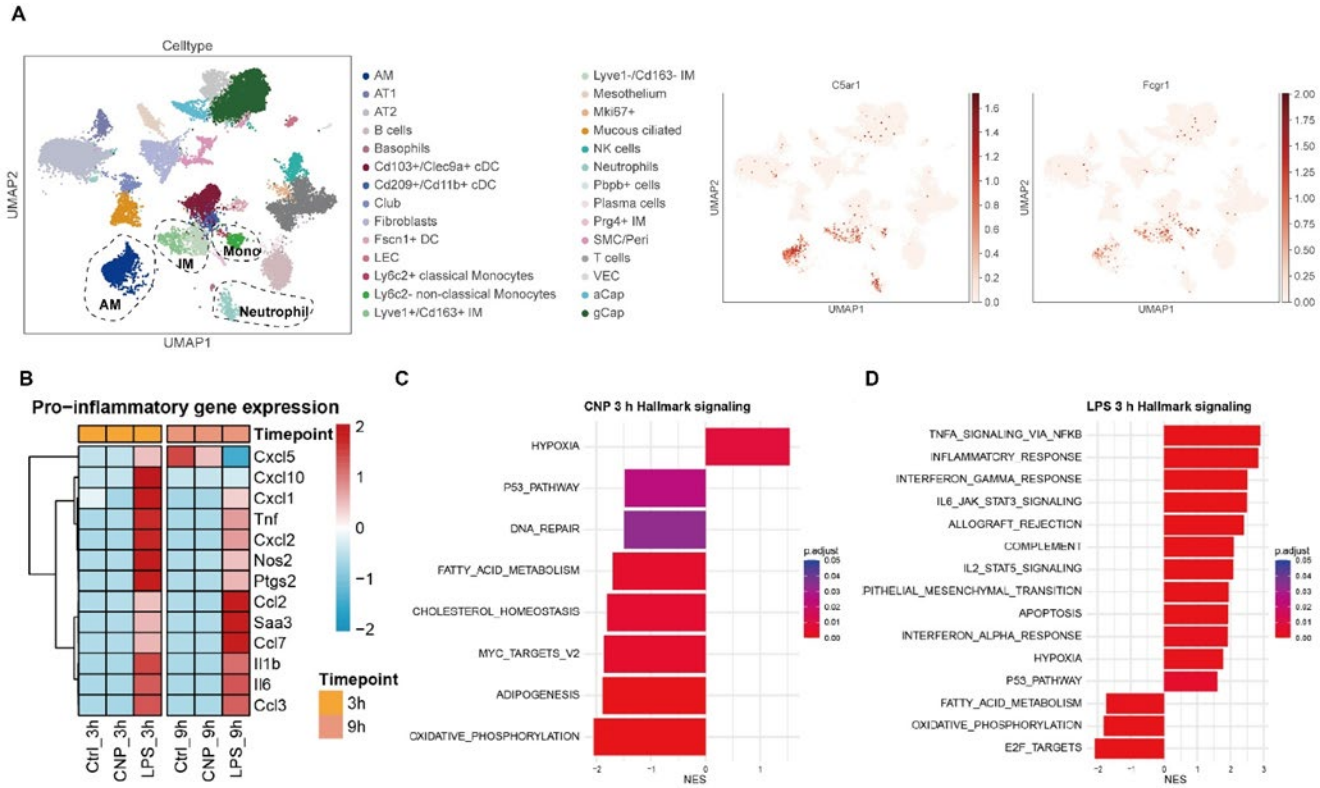


**Fig. S7. Single-cell transcriptomic and pathway analysis reveals inflammatory and signaling responses to CNP and LPS treatment.**

(**A**) Visualization of dimension-reduced single cell transcriptomic data by Uniform Manifold Approximation and Projection (UMAP) reveals different annotated cell types and the expression of genes (GSE185006).

(**B**) Heatmap visualization of classical pro-inflammatory genes induced in CNP and LPS (inflammation control) exposed Ana-1 macrophages after 3 and 9 h.

(**C, D**) Gene Set Enrichment Analysis (GSEA) of Hallmark signaling pathways revealed different pathways for CNP (**C**) and LPS (**D**) after 3 h exposure in Ana-1 macrophages. Result is visualized by bar plot; x-axis: the normalized enrichment score (NES); color code of the bar: the adjusted p-value.

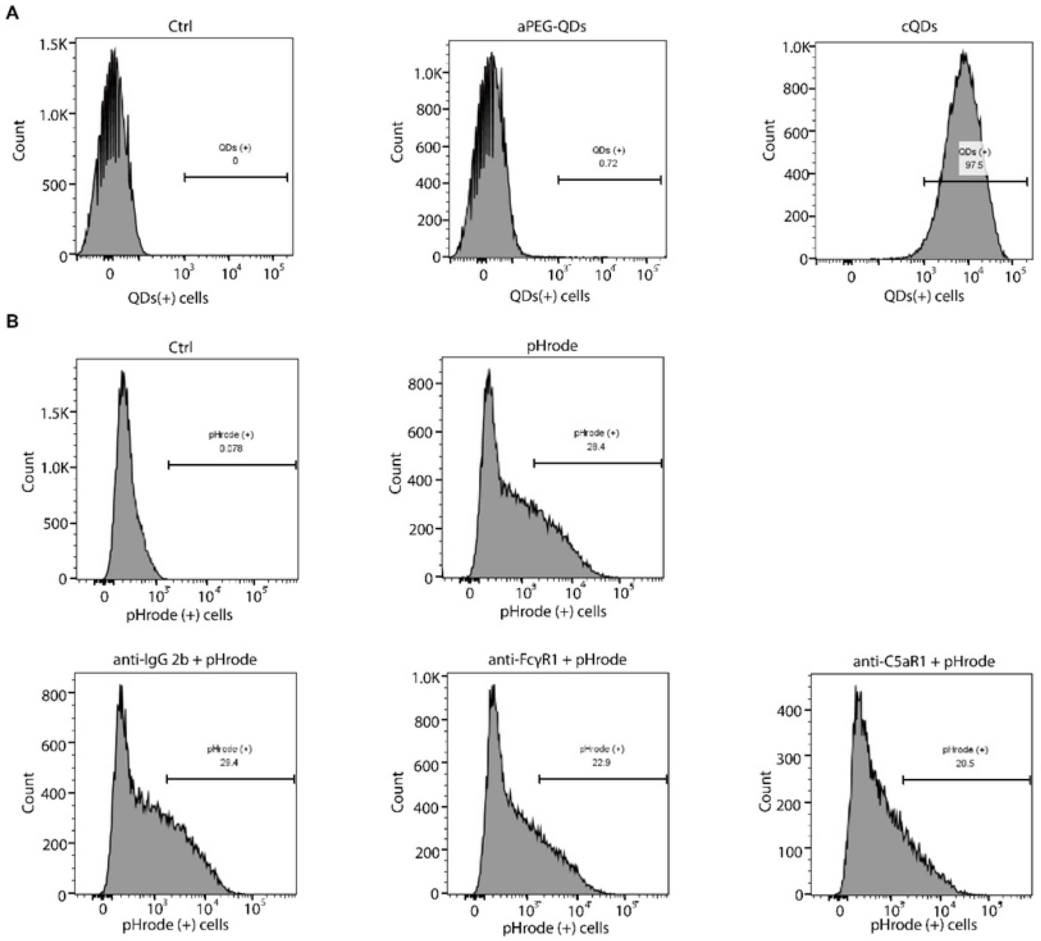


**Fig. S8. Flow cytometry analysis of QD uptake and pHrodo-based phagocytic activity.**
(**A**) Flow cytometry analysis of MH-S macrophage uptake of QDs. cQDs showed high uptake, while aPEG-QDs had minimal uptake.
(**B**) pHrodo-labeled particle uptake (2h) was inhibited by anti-FcγRI and anti-C5aR1 antibodies, indicating receptor involvement in phagocytosis.
